# Standard values of the upper body posture in healthy adults with special regard to age, sex and BMI

**DOI:** 10.1038/s41598-023-27976-8

**Published:** 2023-01-17

**Authors:** D. Ohlendorf, I. Avaniadi, F. Adjami, W. Christian, C. Doerry, V. Fay, V. Fisch, A. Gerez, J. Goecke, U. Kaya, J. Keller, D. Krüger, J. Pflaum, L. Porsch, C. Loewe, B. Scharnweber, P. Sosnov, E. M. Wanke, G. Oremek, H. Ackermann, F. Holzgreve, F. Keil, D. A. Groneberg, C. Maurer-Grubinger

**Affiliations:** 1 Institute of Occupational Medicine, Social Medicine and Environmental Medicine, Goethe-University Frankfurt/Main, Theodor-Stern-Kai 7, Building 9A, 60590 Frankfurt/Main, Germany; 2 Department of Orthodontics, School of Dentistry, Goethe University Frankfurt/Main, Frankfurt, Germany; 3 Institute of Biostatistics and Mathematical Modeling, Goethe-University, Frankfurt/Main, Germany; 4 Institute of Neuroradiology, Goethe-University, Frankfurt/Main, Germany

**Keywords:** Public health, Therapeutics

## Abstract

In order to classify and analyze the parameters of upper body posture in clinical or physiotherapeutic settings, a baseline in the form of standard values with special regard to age, sex and BMI is required. Thus, subjectively healthy men and women aged 21–60 years were measured in this project. The postural parameters of 800 symptom-free male (n = 397) and female (n = 407) volunteers aged 21–60 years (Ø♀: 39.7 ± 11.6, Ø ♂: 40.7 ± 11.5 y) were studied. The mean height of the men was 1.8 ± 0.07 m, with a mean body weight of 84.8 ± 13.1 kg and an average BMI of 26.0 ± 3.534 kg/m^2^. In contrast, the mean height of the women was 1.67 ± 0.06 m, with a mean body weight of 66.5 ± 12.7 kg and an average BMI of 23.9 ± 4.6 kg/m^2^. By means of video rasterstereography, a 3-dimensional scan of the upper back surface was measured when in a habitual standing position. The means or medians, confidence intervals, tolerance ranges, the minimum, 2.5, 25, 50, 75, 97.5 percentiles and the maximum, plus the kurtosis and skewness of the distribution, were calculated for all parameters. Additionally, ANOVA and a factor analyses (sex, BMI, age) were conducted. In both sexes across all age groups, balanced, symmetrical upper body statics were evident. Most strikingly, the females showed greater thoracic kyphosis and lumbar lordosis angles (kyphosis: Ø ♀ 56°, Ø♂ 51°; lordosis: Ø ♀ 49°, Ø♂ 32°) and lumbar bending angles (Ø ♀ 14°, Ø♂ 11°) than the males. The distance between the scapulae was more pronounced in men. These parameters also show an increase with age and BMI, respectively. Pelvic parameters were independent of age and sex. The upper body postures of women and men between the ages of 21 and 60 years were found to be almost symmetrical and axis-conforming with a positive correlation for BMI or age. Consequently, the present body posture parameters allow for comparisons with other studies, as well as for the evaluation of clinical (interim) diagnostics and applications.

## Introduction

Musculoskeletal disorders are a very present problem of our time. In this context, upper body posture is a possible indicator to deduce muscular imbalances and to formulate appropriate preventive measures to counteract or reduce these complaints. Here, sex and age play a major part and are two important aspects, which are also reflected in the posture characterized by certain conditions. With regard to sex differences, these are of physiological but also anatomical genesis. Furthermore, biological functions such as the pain threshold, hormone balance, connective tissue, muscularity or body composition between men and women are also different^1–9^. Related to the latter, men have a wider shoulder distance between the highest point of the inferior scapula angle and a smaller pelvis distance between the spina iliaca posterior superior during habitual standing^5–7,10^. This divergence is due to morphological differences: the pelvic rami and, consequently, the pubic angle are larger in women, as well asthe pelvic tilt angle in general^11,12^. Thus, the pelvis of women is more inclined overall, which in turn causes a slightly greater lordosis in females than in males^5–7,13,14^. Nevertheless, for both sexes the thoracic kyphosis angle is larger than the lumbar lordosis angle^5–7^. Abrisham et al.^14^, using EOS technological analysis of radiographs, showed a mean angle of lordosis of 32.42 ± 6.29° and a mean angle of kyphosis of 43.55 ± 6.44° in approximately 400 male and female subjects aged 18–60 years with low back pain. They could not prove any age-dependent correlations but were able to show sex-dependent correlations of the kyphosis and lordosis angles. While the mean lordosis angle of men was between 31 and 33.5°, that of the women described values between 33.8 and 36.3°. In contrast, Fon et al.^15^ demonstrated age- and gender-dependent changes in the Cobb angle using radiographs. Female subjects aged 20–29 years had a median kyphosis angle of 27°, while for 50–95 year olds the median was 40°; male participants equivalently had median values of 27° and 35°, respectively. The degree of kyphosis increased with age and the increasing rate was higher in females than in males, especially after an age of 40 years. The female breast is a factor to be considered here, with increasing breast size affecting cervical lordosis and sagittal balance, and thus pelvic tilt, rather than thoracic kyphosis and lumbar lordosis^16^. Mammaplasty also has no effect on the spine (radiographs of thoracic kyphosis angle and lumbar lordosis) after 12 months^17^. In addition, breast size also correlates positively with BMI, but not with age^18^.

Drzał-Grabiec et al.^19^ found that from 60 to 90 years, compared to 20–25 years, the angle of trunk inclination and the depth of the thoracic kyphosis increased in both men and women. These authors used the non-invasive photogrammetric Moiré method. Gender-specific age changes in women were found to comprise an increase in the angle of upper thoracic inclination and the angle of shoulder line, together with asymmetry of the scapula position, whereas in men the angle of thoracolumbar inclination and the difference in the distance between the lower scapular angles and the spine increased. In addition, the lordosis angle tended to change from an age of more than 60 years so that the men had a greater lumbar lordosis angle than the women^19^. Furthermore, C2–C7 lordosis demonstrated age-based changes, especially from an age of 55 years onwards^20^. While the distances between the perpendiculars of C2 and C7 did not change, the sagittal angle C2–C7, on the other hand, was found to increase with age^21^.

These exemplary demonstrations of age-related changes in the upper body posture are, among other factors, due to complex physiological processes that occur during the course of a lifetime^22,23^. The aging process includes the decreasing effectiveness of central and peripheral neurons and a decrease in the ratio of bone and muscle tissue mass in relation to total body weight^19,24^. A lower muscle mass implies that a lower muscle strength would be generated. This, in combination with an increasing fragility of the connective tissue, can favor changes in posture and body tension^19,24^. In this context, a change in body composition should also be considered. Here, obesity can lead to structural changes in the spinal shape, especially the thoracolumbar spinal shape, due to increased biomechanical load, for example^25^. Furthermore, the body height may decrease with increasing age due to the lower water content of the intervertebral discs^22^. In addition, surgical procedures can also alter upper body posture. For example, reduction mammaplasty in women improves the alignment of the shoulders as well as the trunk and pelvis, resulting in pain relief in the upper limbs and spine^23^, whereas mastectomy, on the other hand, increases scoliosis^24^. For this purpose, it would be useful, ideally, to be able to document (pathological) changes or their progression from a clinical point of view.

Basically, radiography is irreplaceable here; it records values of spinal changes taken from baseline values to review the parameters of spinal surface calculations^26^. One diagnostic technology is the non-invasive rasterstereography back-surface measurement technique; this allows the 3-dimensional imaging of the back surface via light projections based on triangulation^27–31^. However, with the addition of markers placed on anatomical landmarks, high intraclass correlation coefficients and good Cornbach’s alpha values for intra- and inter-day reliability for all spine parameters can be achieved^32–35^. This applies, among other things, to sagittal evaluation parameters such as the kyphosis and lordosis angles^31,36^. Furthermore, a good inter-tester reliability of 0.979 has been reported^31^. Due to these technical requirements, this procedure of back scanning would be suitable as an alternative method of obtaining an initial impression of the upper body posture or for the interim diagnosis of treatment processes without directly having to take an X-ray image. The advantage here would be that information beyond the spine would be possible, i.e., a complete statement about individual upper body areas (shoulder, spine, pelvis) and not just individual spinal angles. A machine learning-based analysis of rasterstereography images has been used for scoliosis screening and has been proven for the the assignment of such individuals to undergo radiography^37^. It has also been used to document the course of pregnancy, with kyphosis increasing significantly with pregnancy and returning to its pre-birth state after birth, as well as in non-pregnant women. Decriptively similar development is seen in lordosis^38^.

The use of the back scanner has advantages not only in terms of clinical aspects, but also in terms of financial considerations. This is because constantly rising health care costs in industrialized nations has led to insurance companies and healthcare facilities becoming under increasing pressure to evaluate objectively the impairments caused by illness or injury, including spinal complaints or back pain. In this context of objective documentation or intermediate diagnosis, 3-dimensional back scanning is a reliable, non-invasive and cost-effective method. However, reference values are essential for this diagnostic option to be utilized.

Therefore, the aim of this study was to present a superordinate data set of the predominantly working population, subjectively feeling healthy, stratifying both sexes into four age groups (21–30 years, 31–40 years, 41–50 years and 51–60 years)^39^. In addition to age and sex factors, the BMI (height and weight) was also correlated with the upper body parameters.

## Material and methods

The data summarized here have already been published for young men (18–35 years)^5^, men between 31 and 40 years old^9^, middle-aged men (41–50 years)^6^ as well as those between 51 and 60 years old^10^ and for young women (21–30 years)^7^ and middle-aged women (51–60 years)^8^ as part of a larger project^39^. However, the publications of these individual subgroups mainly contain the descriptive presentation of the norm values by means of confidence interval and tolerance range. In this publication, however, the focus is on the overall analysis with special regard on gender- and age-specific differences (as well as BMI). Furthermore, the available data were integrated into an evaluation with a different research question^40^.

### Subjects

All subjects were Caucasian (european) males (n = 397) and females (n = 407) in an age range of 21–60 years who considered themselves as being healthy at the time of measurement. “Healthyˮ means via the self-reporting of no orthopedic or neurological problems, no history of musculoskeletal system injury or surgery, or signs of TMD. All female participants were not pregnant at the time of the measurement or the childbirth had to be at least half a year ago. According to the World Health Organization (WHO) classification^41^, the median weight across all the women was considered normal, while for the men it was pre-obese. Subjects were recruited in the local area of Frankfurt am Main through flyers and word of mouth. No other special inclusion criteria as the beforementioned ones were applied.

With regard to the age group analysis ((1) 21–30 years, (2) 31–40 years, (3) 41–50 years and (4) 51–60 years) of the women, they were of normal weight up to the age of 40 years and from then on pre-adipose (25.2 and 25.0 kg/m^2^, respectively). In contrast, the men were of normal weight up to the age of 30 years and from then on pre-obese with 26.7, 26.7 and 27 kg/m^2^, respectively. All participants were predominantly right-handed. The subjects were equally distributed (age and gender) and their characteristics are summarized in Table 1.

**Table 1:** Biometric distribution of the investigated subjects.

Age group	Age mean	n	Height [m]	Weight [kg]	BMI [kg/m^2^]	Age mean	n	Height [m]	Weight [kg]	BMI [kg/m^2^]
	Women					Men				
All	39.7 ± 11.6	407	1.67 ± 0.06	66.4 ± 12.7	23.9 ± 4.6	40.7 ± 11.5	393	1.8 ± 0.07	84.8 ± 13.1	26 ± 3.5
21–30	25.0 ± 2.7	106	1.69 ± 0.06	60.3 ± 7.8	21.1 ± 2.6	25.2 ± 2.8	92	1.81 ± 0.07	77 ± 10	23.5 ± 2.1
31–40	35.1 ± 3.0	105	1.66 ± 0.06	67.3 ± 13.4	24.3 ± 4.7	35.5 ± 2.9	101	1.8 ± 0.07	86.4 ± 11.6	26.7 ± 3.3
41–50	45.1 ± 3.0	98	1.66 ± 0.06	69.5 ± 14.3	25.2 ± 5	45.6 ± 3.0	100	1.81 ± 0.08	87 ± 12.7	26.7 ± 3.3
51–60	55.1 ± 2.9	98	1.66 ± 0.06	69.2 ± 12.3	25 ± 4.6	55.3 ± 2.8	100	1.8 ± 0.08	88.1 ± 14.6	27 ± 3.9

Before the study was conducted, each participant was required to sign a written consent and to complete a medical history form and an amnesis questionnaire (Centre for Dental, Oral and Maxillofacial Medicine of the Goethe University Frankfurt am Main^15^). The latter included questions on general diseases such as osteoporosis, diabetes mellitus, pain in the joints in general, noises in the ears as well as complaints in the temporomandibular joint. The test persons were also asked about possible accident damage to the mouth, jaw and face areas and to the musculoskeletal system. Furthermore, they would be asked whether they participate in sports (rarely/never vs. yes).

Other factors may also influence upper body statics, such as occupation (asymmetric/symmetrical, repetitive work activities), or compensatory movements, among others. However, these were not addressed in this analysis. After being fully informed about the risk-free nature of the evaluation and the aims of this study research, each subject voluntarily signed a consent form. The study was in accordance with the 1964 Helsinki Declaration and its later amendments and was approved by the local medical ethics committee of the Faculty of Medical Science, Goethe University Frankfurt, Germany (approval No. 303/16).

### Patient and public involvement

No patients were involved. All volunteers were healthy and informed about the study design before giving written informed consent.

### Measurement system

The clear benefit of a 3-dimensional scan to a 2-dimensional photography^42^ or video analysis^43,44^ is the depth information calculated from the projected lines onto a curvature. With progress in image reconstruction algorithms, the surface parameters of the upper body can be calculated^5,7,27,45^.

The contactless, light-optical back scanner “ABW-BodyMapper” (ABW GmbH, Frickenhausen/Germany) was used to measure the 3-dimensional upper body posture by means of video rasterstereography. The depth resolution of the generated resultant image was 1/100 mm and the maximum image frequency was 50 frames/sec. During recording the measurement errors should be < 1 mm and the measurement accuracy less than 0.5 mm according to the manufacturer. Each test person had six anatomical landmarks (light-reflecting markers of 1 cm in diameter) attached to their back in order to achieve optimum measurements of the back surface. Formulae or algorithms used to calculate the evaluation parameters have been published by Ohlendorf et al.^5,6,39^. This method has a high correlation for intra- and inter-day reliability for all spine parameters^32–35^, especially in relation to the sagittal evaluation parameters such as the kyphosis or lordosis angles^31,36^. In addition, a good inter-tester reliability of 0.979, despite the setting of anatomical landmarks on the back, has been reported^31^.

The coefficients observed for the inter-day and inter-week were slightly lower than for repeated measurements on the same day in this study. The standard error of the mean was less than 1.5° or 1.5 mm, except for the trunk inclination^35^.

### Measure protocol

The subjects stood barefoot in a habitual posture, approximately 90 cm in front of the back scanner, with their arms hung loosely, looking horizontally at the opposite wall. In order to obtain reproducible values, three repeated measurements were performed within 2 min.

### Evaluation of parameters

**Figure 1:**
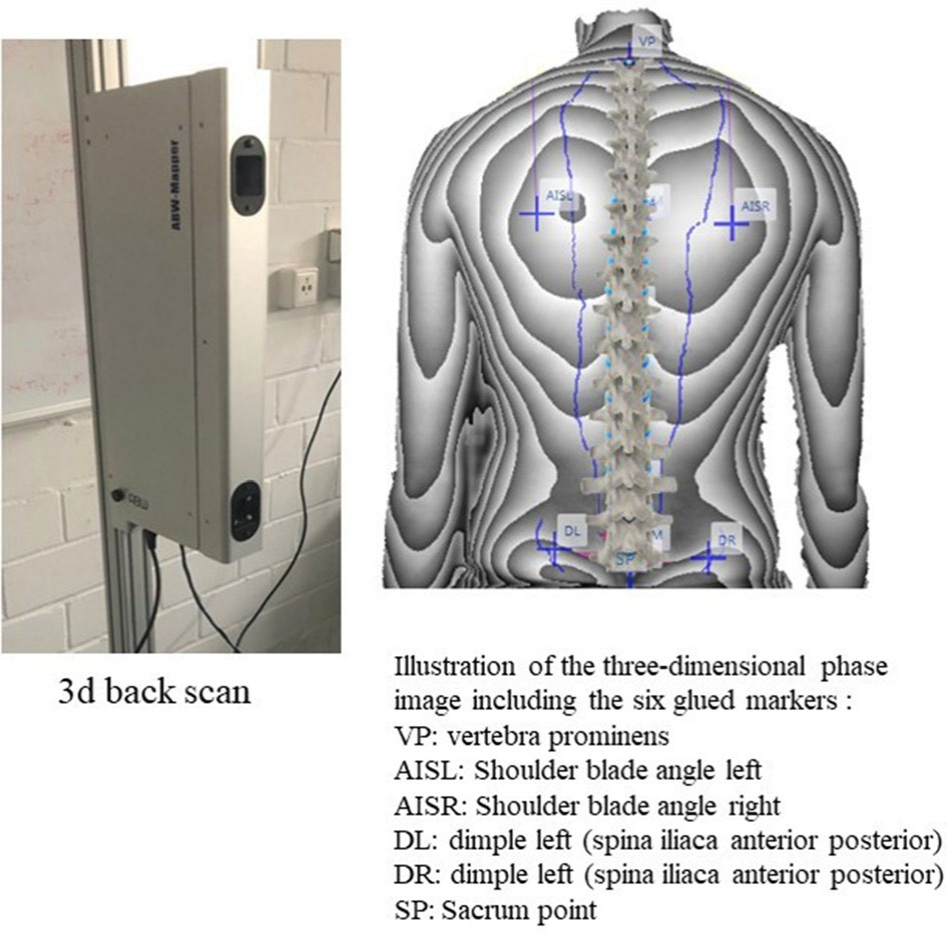
Back scan and the six markers set on the bare back including their names.

All parameters were divided into three categories according to the anatomical topography: (a) the markers of the spinal column variables ranged from the 7th cervical vertebra to the Rima Ani, (b) the shoulder variables enclosed markers on the shoulder blade, and (c) the pelvic variables were derived from the marker positions on the left and right SIPS (spina ilica posterior superior). The precise placements of the six landmarks are illustrated in Fig. 1. The definition and interpretation of each parameter are listed corresponding to each parameter in Tables 2,3,4. The definitions of the parameters are specified and defined accordingly by the manufacturer and are, therefore, adopted in the following evaluations.

**Table 2:** Median, confidence interval (lower/upper limit), minimum (Min), percentile (P) 2.5, quartiles 1 and 3, percentile 97.5, maximum (Max), kurtosis and skewness of the spine parameters.

Age group	Median	Confidence Interval-Lower limit	Confidence Interval-Upper limit	Min	P 2.5	Quartile 1	Quartile 3	P 97.5	Max	Kurtosis	Skew-ness	Median	Confidence Interval-Lower limit	Confidence Interval-Upper limit	Min	P 2.5	Quartile 1	Quartile 3	P 97.5	Max	Kurtosis	Skew-ness
	Women											Men										
Trunk length D (Spatial distance between the markers C7 and middle of the PSIS-marker) (mm)																						
21–60	**453.51**	**451.06**	**455.96**	**374.71**	**404.85**	**436.73**	**471.06**	**503.54**	**525.32**	**3.03**	− **0.05**	*488.76*	*485.67*	*491.85*	*373.67*	*424.15*	*469.24*	*508.15*	*544.42*	*596.68*	*3.82*	− *0.02*
21–30	**459.68**	**455.00**	**464.35**	**413.10**	**416.79**	**444.06**	**478.22**	**508.82**	**513.26**	**2.32**	**0.17**	*475.17*	*469.34*	*481.00*	*373.67*	*421.07*	*461.33*	*498.50*	*531.60*	*536.00*	*3.98*	− *0.55*
31–40	**454.13**	**449.14**	**459.12**	**374.71**	**396.39**	**438.83**	**467.05**	**499.12**	**525.32**	**3.89**	− **0.32**	*476.95*	*471.04*	*482.87*	*405.02*	*419.75*	*452.48*	*490.26*	*539.93*	*555.56*	*2.88*	*0.13*
41–50	**443.67**	**438.73**	**448.61**	**376.32**	**400.14**	**432.59**	**465.25**	**494.28**	**495.51**	**2.74**	− **0.05**	*503.50*	*497.96*	*509.04*	*414.26*	*450.61*	*483.27*	*516.38*	*564.02*	*595.83*	*4.35*	*0.31*
51–60	**450.95**	**446.10**	**455.80**	**396.94**	**410.30**	**434.81**	**467.31**	**503.77**	**510.89**	**2.62**	**0.09**	*499.57*	*493.75*	*505.38*	*402.19*	*437.38*	*479.13*	*514.00*	*552.13*	*596.68*	*4.67*	*0.09*
Trunk length S (Spatial distance between the markers at C7 and Rima Ani) (mm)																						
21–60	**495.96**	**493.20**	**498.72**	**392.83**	**436.95**	**476.55**	**515.16**	**550.20**	**570.47**	**3.03**	− **0.14**	*533.77*	*530.70*	*536.84*	*428.67*	*472.78*	*512.55*	*553.29*	*597.92*	*648.55*	*3.57*	*0.11*
21–30	**506.64**	**501.75**	**511.53**	**457.57**	**466.01**	**491.83**	**529.57**	**555.38**	**570.47**	**2.30**	**0.19**	*528.17*	*521.98*	*534.35*	*428.67*	*469.27*	*508.33*	*547.00*	*584.20*	*589.00*	*3.49*	− *0.29*
31–40	**498.62**	**493.31**	**503.94**	**411.43**	**436.26**	**483.06**	**517.02**	**548.78**	**559.88**	**3.38**	− **0.42**	*526.03*	*520.50*	*531.57*	*467.23*	*479.25*	*503.40*	*546.37*	*588.34*	*606.26*	*2.87*	*0.32*
41–50	**482.80**	**477.51**	**488.09**	**392.83**	**436.70**	**469.76**	**501.68**	**532.85**	**538.90**	**3.41**	− **0.24**	*545.35*	*538.97*	*551.73*	*455.28*	*497.04*	*521.47*	*563.46*	*609.27*	*648.55*	*3.50*	*0.28*
51–60	**487.53**	**482.09**	**492.96**	**426.76**	**431.16**	**469.87**	**505.24**	**535.21**	**554.55**	**2.63**	− **0.07**	*536.56*	*530.54*	*542.57*	*443.95*	*467.38*	*514.38*	*553.84*	*593.38*	*618.87*	*3.97*	− *0.16*
Sagittal trunk decline ( Inclination of the trunk length D marked line from the perpendicular to the sagittal plane) (°)																						
21–60	− **3.29**	− **3.58**	− **3.01**	− **12.33**	− **10.38**	− **5.34**	− **1.56**	**1.80**	**3.58**	**3.25**	− **0.42**	− *3.24*	− *3.51*	− *2.97*	− *11.96*	− *8.38*	− *5.15*	− *1.47*	*1.85*	*6.40*	*3.15*	*0.05*
21–30	− **3.41**	− **3.87**	− **2.94**	− **10.35**	− **8.06**	− **4.99**	− **1.55**	**2.07**	**3.04**	**3.21**	**0.03**	− *4.00*	− *4.49*	− *3.51*	− *10.00*	− *8.20*	− *5.33*	− *2.33*	*1.07*	*2.67*	*3.09*	*0.05*
31–40	− **3.29**	− **3.85**	− **2.74**	− **11.59**	− **10.25**	− **5.41**	− **1.75**	**1.56**	**3.58**	**3.27**	− **0.43**	− *2.78*	− *3.35*	− *2.20*	− *8.70*	− *7.52*	− *4.98*	− *0.62*	*2.88*	*6.40*	*2.86*	*0.28*
41–50	− **3.04**	− **3.69**	− **2.39**	− **12.33**	− **11.24**	− **5.55**	− **1.49**	**2.41**	**2.93**	**3.10**	− **0.48**	− *3.12*	− *3.58*	− *2.66*	− *9.74*	− *8.25*	− *4.89*	− *1.68*	*0.91*	*1.39*	*2.72*	− *0.25*
51–60	− **3.78**	− **4.40**	− **3.15**	− **11.89**	− **10.97**	− **5.74**	− **1.68**	**1.68**	**2.36**	**2.81**	− **0.45**	− *3.52*	− *4.09*	− *2.94*	− *11.96*	− *8.72*	− *5.63*	− *1.55*	*1.78*	*3.30*	*2.86*	− *0.17*
Frontal trunk decline (Inclination of the trunk length D marked line from the perpendicular to the frontal plane) (°)																						
21–60	− **0.17**	− **0.29**	− **0.04**	− **3.99**	− **2.56**	− **0.98**	**0.71**	**2.34**	**3.26**	**3.04**	− **0.03**	− *0.16*	− *0.29*	− *0.03*	− *5.27*	− *3.03*	− *1.00*	*0.66*	*2.33*	*4.13*	*3.70*	− *0.30*
21–30	− **0.34**	− **0.58**	− **0.10**	− **3.82**	− **3.01**	− **1.20**	**0.39**	**1.87**	**2.15**	**2.74**	− **0.28**	*0.33*	*0.11*	*0.56*	− *3.00*	− *1.73*	*0.00*	*1.00*	*2.33*	*2.33*	*2.98*	− *0.29*
31–40	− **0.10**	− **0.32**	**0.12**	− **2.41**	− **2.22**	− **1.10**	**0.61**	**2.16**	**3.13**	**2.86**	**0.20**	− *0.34*	− *0.59*	− *0.09*	− *4.22*	− *3.37*	− *1.36*	*0.30*	*1.53*	*2.73*	*3.43*	− *0.42*
41–50	**0.23**	− **0.01**	**0.48**	− **2.47**	− **1.76**	− **0.59**	**1.17**	**2.81**	**2.89**	**2.32**	**0.18**	− *0.40*	− *0.65*	− *0.16*	− *3.40*	− *2.74*	− *1.18*	*0.20*	*2.41*	*3.23*	*3.51*	*0.13*
51–60	− **0.29**	− **0.56**	− **0.03**	− **3.99**	− **3.60**	− **1.26**	**0.71**	**2.27**	**3.26**	**3.41**	− **0.15**	− *0.34*	− *0.63*	− *0.05*	− *5.27*	− *3.19*	− *1.25*	*0.67*	*2.55*	*4.13*	*4.26*	− *0.36*
Axis decline ( Deviation of the line of the area marked by the trunk length D line of the 90° rotated distance between PSIS left and PSIS right) (°)																						
21–60	− **0.62**	− **0.84**	− **0.40**	− **8.58**	− **4.45**	− **1.85**	**0.88**	**4.43**	**11.47**	**4.91**	**0.59**	− *0.67*	− *0.90*	− *0.44*	− *6.98*	− *5.15*	− *2.09*	*0.95*	*4.00*	*6.44*	*2.79*	*0.05*
21–30	**0.05**	− **0.40**	**0.50**	− **5.48**	− **3.57**	− **1.58**	**1.56**	**5.71**	**6.76**	**3.12**	**0.38**	− *0.33*	− *0.81*	*0.15*	− *5.00*	− *4.47*	− *2.00*	*1.33*	*4.13*	*5.00*	*2.47*	*0.13*
31–40	− **0.76**	− **1.15**	− **0.37**	− **5.43**	− **4.20**	− **2.15**	**0.76**	**3.69**	**4.22**	**2.70**	**0.20**	− *0.98*	− *1.40*	− *0.57*	− *5.42*	− *4.64*	− *2.59*	*0.36*	*3.20*	*4.10*	*2.63*	*0.20*
41–50	− **1.22**	− **1.57**	− **0.88**	− **4.60**	− **4.21**	− **1.91**	**0.19**	**2.70**	**3.94**	**3.20**	**0.51**	− *0.96*	− *1.42*	− *0.51*	− *6.98*	− *5.66*	− *2.36*	*0.45*	*4.16*	*6.44*	*3.52*	*0.28*
51–60	− **0.53**	− **1.07**	**0.02**	− **8.58**	− **5.38**	− **1.80**	**1.01**	**4.57**	**11.47**	**6.04**	**0.65**	− *0.05*	− *0.54*	*0.44*	− *6.27*	− *5.90*	− *1.84*	*1.55*	*4.17*	*4.80*	*2.75*	− *0.42*
Thoracic bending angle (Deviation of the distance C7 – Kyphosis Apex from the perpendicular) (°)																						
21–60	**13.90**	**13.50**	**14.30**	**5.14**	**7.13**	**10.87**	**16.75**	**23.03**	**29.18**	**3.36**	**0.52**	*16.23*	*15.84*	*16.62*	*5.84*	*9.48*	*13.60*	*18.74*	*24.63*	*28.53*	*3.04*	*0.30*
21–30	**13.85**	**13.14**	**14.57**	**6.71**	**7.04**	**11.03**	**16.52**	**21.13**	**25.20**	**2.68**	**0.32**	*16.17*	*15.47*	*16.87*	*9.33*	*10.40*	*14.17*	*18.33*	*24.40*	*24.67*	*2.86*	*0.31*
31–40	**13.75**	**13.01**	**14.48**	**5.30**	**8.30**	**11.85**	**16.17**	**23.40**	**28.99**	**5.47**	**0.92**	*15.78*	*15.02*	*16.54*	*5.84*	*7.78*	*13.03*	*18.22*	*23.24*	*27.92*	*3.48*	*0.09*
41–50	**12.81**	**11.84**	**13.77**	**5.14**	**6.71**	**10.55**	**17.19**	**24.65**	**29.18**	**3.06**	**0.68**	*16.06*	*15.27*	*16.84*	*8.90*	*9.96*	*12.97*	*18.53*	*24.61*	*28.53*	*3.05*	*0.60*
51–60	**14.45**	**13.62**	**15.28**	**6.68**	**7.22**	**11.47**	**17.01**	**22.43**	**24.17**	**2.36**	**0.08**	*17.26*	*16.44*	*18.07*	*7.86*	*10.25*	*14.91*	*20.09*	*26.04*	*27.02*	*2.63*	*0.15*
Kyphosis angle (Angle between the upper turning point at C7 and the thoracolumbar inflection point) (°)																						
21–60	**56.28**	**54.74**	**57.82**	**19.57**	**31.85**	**46.73**	**67.29**	**92.48**	**108.00**	**3.11**	**0.45**	*51.29*	*50.32*	*52.25*	*22.67*	*31.55*	*44.30*	*57.02*	*70.13*	*84.54*	*3.14*	*0.07*
21–30	**52.33**	**50.03**	**54.62**	**20.95**	**29.18**	**45.18**	**59.70**	**75.24**	**79.06**	**2.75**	− **0.02**	*45.17*	*43.18*	*47.16*	*22.67*	*27.60*	*40.33*	*51.00*	*67.40*	*71.33*	*3.19*	*0.23*
31–40	**59.53**	**56.50**	**62.56**	**24.78**	**33.14**	**48.29**	**67.43**	**91.95**	**108.00**	**3.46**	**0.61**	*52.90*	*51.10*	*54.71*	*32.30*	*35.71*	*47.10*	*58.40*	*71.54*	*78.58*	*3.05*	*0.20*
41–50	**58.70**	**55.26**	**62.14**	**20.82**	**30.60**	**46.27**	**72.86**	**96.02**	**99.75**	**2.46**	**0.27**	*52.41*	*50.52*	*54.30*	*28.47*	*32.50*	*44.21*	*58.33*	*70.25*	*73.84*	*2.60*	− *0.04*
51–60	**59.68**	**56.37**	**62.98**	**19.57**	**32.78**	**48.43**	**74.32**	**99.37**	**103.55**	**2.78**	**0.28**	*53.60*	*51.90*	*55.31*	*34.42*	*38.25*	*48.24*	*58.86*	*72.04*	*84.54*	*3.99*	*0.47*
Lumbar bending angle (Deviation of the distance Kyphosis Apex – Lordosis Apex from the perpendicular) (°)																						
21–60	**14.25**	**13.85**	**14.66**	**5.35**	**8.29**	**11.55**	**17.20**	**23.57**	**32.62**	**4.33**	**0.83**	*10.56*	*10.26*	*10.87*	*3.00*	*5.33*	*8.62*	*12.57*	*16.56*	*27.58*	*4.91*	*0.56*
21–30	**13.16**	**12.43**	**13.89**	**5.35**	**7.92**	**10.81**	**16.15**	**22.70**	**23.72**	**2.90**	**0.54**	*10.00*	*9.31*	*10.69*	*3.00*	*4.27*	*8.00*	*12.00*	*18.20*	*21.33*	*3.67*	*0.50*
31–40	**14.73**	**13.94**	**15.52**	**5.54**	**8.52**	**12.35**	**17.97**	**22.98**	**32.62**	**5.14**	**0.80**	*11.20*	*10.66*	*11.75*	*5.48*	*6.32*	*8.79*	*12.72*	*16.19*	*19.21*	*2.86*	*0.33*
41–50	**14.27**	**13.33**	**15.20**	**6.75**	**8.45**	**12.26**	**17.57**	**27.61**	**29.36**	**3.85**	**1.01**	*10.62*	*10.09*	*11.14*	*4.39*	*5.76*	*9.05*	*12.44*	*15.26*	*15.76*	*2.39*	− *0.11*
51–60	**14.45**	**13.67**	**15.22**	**7.22**	**8.27**	**11.81**	**17.00**	**21.68**	**30.03**	**4.40**	**0.67**	*10.78*	*10.10*	*11.46*	*4.54*	*4.94*	*8.78*	*13.15*	*17.07*	*27.58*	*6.91*	*1.06*
Lordosis angle (Angle between the lower inflection point at the center of the PSIS marker and the thoracolumbar turning point) (°)																						
21–60	**49.02**	**47.49**	**50.55**	**4.52**	**23.80**	**38.84**	**59.28**	**85.57**	**105.69**	**3.50**	**0.41**	*31.96*	*31.08*	*32.83*	*1.89*	*14.89*	*26.39*	*37.77*	*48.71*	*65.84*	*3.38*	− *0.07*
21–30	**46.08**	**43.70**	**48.46**	**16.52**	**21.33**	**37.10**	**55.59**	**71.56**	**75.87**	**2.60**	**0.05**	*30.67*	*28.83*	*32.51*	*7.00*	*10.07*	*24.50*	*35.67*	*46.47*	*49.00*	*2.99*	− *0.25*
31–40	**47.47**	**44.51**	**50.43**	**4.52**	**21.73**	**39.77**	**58.79**	**77.71**	**81.66**	**3.13**	− **0.08**	*31.72*	*30.24*	*33.19*	*18.07*	*19.29*	*27.13*	*36.33*	*47.52*	*56.34*	*3.07*	*0.37*
41–50	**50.54**	**46.97**	**54.10**	**5.22**	**18.15**	**38.19**	**62.50**	**94.10**	**105.69**	**3.47**	**0.44**	*32.66*	*30.94*	*34.38*	*11.85*	*16.76*	*26.37*	*38.46*	*49.34*	*50.98*	*2.50*	− *0.08*
51–60	**51.18**	**47.90**	**54.47**	**24.85**	**26.08**	**40.41**	**61.20**	**88.88**	**95.96**	**2.88**	**0.58**	*33.35*	*31.43*	*35.28*	*1.89*	*14.58*	*27.56*	*40.29*	*49.62*	*65.84*	*4.00*	− *0.19*
Standard deviation of lateral deviation (Root mean squared deviation of the median line of the distance C7 – Center of the PSIS Marker) (mm)																						
21–60	**3.84**	**3.63**	**4.04**	**0.79**	**1.31**	**2.64**	**5.34**	**9.54**	**13.00**	**4.02**	**1.00**	*3.77*	*3.55*	*4.00*	*0.69*	*1.30*	*2.67*	*5.33*	*9.60*	*14.00*	*4.12*	*1.09*
21–30	**3.78**	**3.45**	**4.11**	**0.95**	**1.21**	**2.55**	**5.07**	**8.18**	**8.78**	**3.08**	**0.66**	*3.83*	*3.34*	*4.33*	*1.00*	*1.33*	*2.67*	*5.17*	*10.07*	*14.00*	*5.04*	*1.33*
31–40	**4.13**	**3.69**	**4.58**	**1.32**	**1.55**	**2.97**	**6.08**	**9.98**	**13.00**	**3.85**	**1.01**	*4.70*	*4.22*	*5.19*	*1.21*	*1.54*	*2.89*	*6.51*	*10.87*	*11.89*	*3.02*	*0.75*
41–50	**3.77**	**3.33**	**4.20**	**1.00**	**1.31**	**2.95**	**5.89**	**9.80**	**10.06**	**2.93**	**0.75**	*3.28*	*2.87*	*3.69*	*0.69*	*1.30*	*2.43*	*4.63*	*8.94*	*12.10*	*4.98*	*1.35*
51–60	**3.62**	**3.22**	**4.01**	**0.79**	**1.25**	**2.31**	**4.80**	**8.12**	**11.62**	**5.08**	**1.23**	*3.81*	*3.41*	*4.21*	*1.03*	*1.14*	*2.69*	*5.16*	*9.11*	*10.36*	*3.43*	*0.84*
Standard deviation of rotation (Root mean square deviation of surface rotation of the median line (torsion of the spinous processes of the spine) (°)																						
21–60	**4.18**	**3.96**	**4.40**	**1.22**	**1.53**	**2.94**	**5.98**	**10.31**	**14.11**	**4.27**	**1.08**	*3.54*	*3.32*	*3.76*	*0.73*	*1.44*	*2.67*	*5.05*	*10.26*	*15.94*	*6.08*	*1.49*
21–30	**4.66**	**4.16**	**5.15**	**1.85**	**2.05**	**3.25**	**6.82**	**12.25**	**14.11**	**4.28**	**1.13**	*3.67*	*3.28*	*4.06*	*1.00*	*1.67*	*2.67*	*4.67*	*9.53*	*10.33*	*5.23*	*1.43*
31–40	**3.99**	**3.58**	**4.41**	**1.22**	**1.80**	**2.93**	**6.10**	**9.65**	**11.04**	**3.05**	**0.76**	*3.41*	*2.90*	*3.92*	*0.73*	*1.28*	*2.38*	*5.16*	*11.28*	*15.94*	*7.14*	*1.76*
41–50	**4.33**	**3.90**	**4.76**	**1.47**	**1.53**	**2.85**	**5.30**	**10.18**	**11.69**	**4.15**	**1.02**	*3.41*	*3.05*	*3.77*	*0.99*	*1.12*	*2.59*	*4.74*	*8.22*	*10.12*	*3.98*	*1.04*
51–60	**3.81**	**3.37**	**4.25**	**1.22**	**1.36**	**2.45**	**5.24**	**10.15**	**11.49**	**4.13**	**1.13**	*3.89*	*3.44*	*4.35*	*1.29*	*1.50*	*2.87*	*5.81*	*10.78*	*11.24*	*3.71*	*1.05*

All data are color-coded by sex (women = Bold; men = Italic). For each sex, four age groups (21–30, 31–40, 41–50, 51–60) and all age groups are shown combined.

**Table 3:** Median, confidence interval (lower/upper limit), minimum (Min), percentile (P) 2.5, quartiles 1 and 3, percentile 97.5, maximum (Max), kurtosis and skewness of the shoulder parameters.

Age group	Median	Confidence Interval-Lower limit	Confidence Interval-Upper limit	Min	P 2.5	Quartile1	Quartile3	p 97.5	Max	Kurtosis	Skew-ness	Median	Confidence Interval-Lower limit	Confidence Interval-Upper limit	Min	P 2.5	Quartile1	Quartile3	p 97.5	Max	Kurtosis	Skew-ness
		WOMEN										MEN										
Scapula distance (Distance between the left (AISL) and the lower right scapula angle (AISR)) (mm)																						
21–60	**161.30**	**159.09**	**163.52**	**82.08**	**120.51**	**145.12**	**177.44**	**206.93**	**229.79**	**3.03**	**0.11**	*188.09*	*185.51*	*190.68*	*118.17*	*142.62*	*171.25*	*206.05*	*245.63*	*292.17*	*3.52*	*0.35*
21–30	**149.77**	**145.90**	**153.64**	**82.08**	**111.28**	**138.25**	**163.39**	**187.10**	**198.60**	**3.41**	− **0.23**	*177.67*	*172.69*	*182.64*	*122.33*	*134.60*	*162.00*	*192.33*	*233.87*	*249.33*	*3.21*	*0.35*
31–40	**158.03**	**154.00**	**162.06**	**109.79**	**121.72**	**141.37**	**169.32**	**199.90**	**216.55**	**3.01**	**0.35**	*185.05*	*180.38*	*189.72*	*118.17*	*140.17*	*166.29*	*203.41*	*225.29*	*248.18*	*2.93*	− *0.19*
41–50	**176.61**	**172.48**	**180.74**	**126.86**	**138.00**	**161.08**	**188.44**	**217.16**	**229.79**	**2.90**	**0.11**	*187.46*	*181.57*	*193.36*	*142.43*	*148.68*	*174.15*	*213.17*	*266.03*	*292.17*	*3.46*	*0.80*
51–60	**164.03**	**159.72**	**168.33**	**116.54**	**125.11**	**150.67**	**179.73**	**204.18**	**226.04**	**2.76**	**0.10**	*198.40*	*194.01*	*202.79*	*146.91*	*148.91*	*184.68*	*212.75*	*239.98*	*267.54*	*3.32*	− *0.04*
Scapula height (Height difference between the points AISL and AISR) (mm)																						
21–60	− **1.95**	− **2.76**	− **1.15**	− **30.91**	− **16.66**	− **6.58**	**3.74**	**14.28**	**28.46**	**3.63**	− **0.05**	− *2.00*	− *2.76*	− *1.24*	− *25.50*	− *17.60*	− *6.46*	*3.33*	*14.10*	*24.04*	*3.64*	*0.06*
21–30	− **2.32**	− **4.35**	− **0.28**	− **30.91**	− **23.33**	− **7.85**	**6.57**	**19.64**	**28.46**	**3.20**	**0.05**	− *2.50*	− *4.20*	− *0.80*	− *25.50*	− *22.20*	− *6.83*	*2.17*	*14.40*	*16.33*	*3.42*	− *0.26*
31–40	− **1.50**	− **2.84**	− **0.17**	− **19.16**	− **15.25**	− **5.68**	**4.09**	**11.41**	**12.09**	**2.49**	− **0.22**	− *4.35*	− *5.74*	− *2.97*	− *19.79*	− *18.66*	− *8.36*	*1.21*	*10.31*	*14.39*	*2.79*	*0.19*
41–50	− **3.54**	− **5.00**	− **2.09**	− **25.31**	− **15.08**	− **7.58**	**0.98**	**13.78**	**15.36**	**3.37**	**0.18**	− *1.15*	− *2.32*	*0.02*	− *12.67*	− *11.82*	− *4.19*	*3.06*	*11.22*	*12.23*	*2.60*	*0.16*
51–60	**0.03**	− **1.46**	**1.51**	− **27.96**	− **17.03**	− **4.53**	**4.91**	**12.59**	**14.20**	**4.07**	− **0.64**	− *0.64*	− *2.35*	*1.07*	− *23.84*	− *17.45*	− *5.43*	*4.89*	*22.17*	*24.04*	*3.76*	*0.18*
Scapula rotation (Rotation of the distance AISL—AISR in the transversal plane) (°)																						
21–60	**1.75**	**1.42**	**2.09**	− **10.77**	− **5.46**	− **0.78**	**3.88**	**8.04**	**11.49**	**3.21**	− **0.29**	*1.00*	*0.69*	*1.31*	− *10.18*	− *5.88*	− *1.04*	*3.02*	*6.93*	*11.31*	*3.42*	− *0.21*
21–30	**3.08**	**2.47**	**3.69**	− **10.77**	− **3.19**	**1.40**	**4.68**	**8.79**	**11.49**	**5.58**	− **0.67**	*1.00*	*0.35*	*1.65*	− *6.67*	− *6.00*	− *1.67*	*2.33*	*7.40*	*8.67*	*2.90*	− *0.04*
31–40	**0.93**	**0.29**	**1.57**	− **6.03**	− **5.51**	− **1.05**	**3.52**	**7.11**	**9.49**	**2.51**	**0.01**	*0.57*	− *0.04*	*1.19*	− *6.41*	− *5.32*	− *1.30*	*2.59*	*6.70*	*8.16*	*2.70*	*0.11*
41–50	**0.95**	**0.23**	**1.67**	− **9.66**	− **7.90**	− **1.87**	**3.18**	**6.89**	**7.72**	**2.79**	− **0.39**	*1.23*	*0.67*	*1.78*	− *4.77*	− *4.06*	− *0.42*	*3.17*	*6.79*	*11.31*	*3.57*	*0.40*
51–60	**1.48**	**0.84**	**2.12**	− **7.66**	− **4.06**	− **0.93**	**3.32**	**8.22**	**8.97**	**2.96**	− **0.03**	*1.12*	*0.40*	*1.83*	− *10.18*	− *7.73*	− *1.12*	*3.35*	*7.19*	*8.46*	*3.59*	− *0.72*
Scapula angle left (Angle of the compensation line applied to the shoulders to the horizontal. The center of the compensation line is specified vertically above AISL) (°)																						
21–60	**27.40**	**26.29**	**28.52**	**3.97**	**16.58**	**23.51**	**31.78**	**69.69**	**76.55**	**8.08**	**2.11**	*26.22*	*25.53*	*26.92*	*5.67*	*16.92*	*23.16*	*29.89*	*42.78*	*71.23*	*11.13*	*1.72*
21–30	**28.54**	**26.66**	**30.42**	**13.32**	**17.44**	**24.39**	**32.64**	**59.90**	**69.56**	**7.61**	**1.83**	*26.00*	*24.57*	*27.43*	*5.67*	*13.18*	*23.67*	*29.08*	*37.70*	*65.00*	*13.70*	*1.67*
31–40	**27.67**	**25.85**	**29.50**	**15.28**	**16.43**	**24.21**	**31.70**	**54.73**	**60.07**	**5.20**	**1.51**	*26.60*	*25.18*	*28.02*	*11.18*	*16.93*	*23.48*	*29.70*	*40.19*	*71.23*	*16.28*	*2.54*
41–50	**26.14**	**23.14**	**29.14**	**3.97**	**14.77**	**22.05**	**29.53**	**74.21**	**76.55**	**6.38**	**2.00**	*26.49*	*24.95*	*28.02*	*14.35*	*17.45*	*22.94*	*30.59*	*48.43*	*58.78*	*6.86*	*1.61*
51–60	**27.37**	**25.07**	**29.67**	**16.83**	**17.25**	**23.09**	**31.93**	**72.99**	**74.92**	**9.34**	**2.41**	*25.87*	*24.66*	*27.08*	*6.43*	*15.65*	*22.40*	*29.33*	*37.91*	*50.29*	*5.26*	*0.36*
Scapula angle right (Angle of the compensation line applied to the shoulders to the horizontal. The center of the compensation line is specified vertically above AISR) (°)																						
21–60	**28.51**	**27.49**	**29.53**	**0.94**	**15.83**	**25.15**	**34.42**	**60.63**	**75.89**	**6.82**	**1.49**	*28.32*	*27.63*	*29.01*	*9.99*	*15.80*	*24.49*	*31.48*	*46.71*	*65.33*	*7.67*	*1.25*
21–30	**31.17**	**28.62**	**33.71**	**3.71**	**12.43**	**23.85**	**38.43**	**72.26**	**75.89**	**4.82**	**1.04**	*29.00*	*27.49*	*30.51*	*12.33*	*16.91*	*26.33*	*31.92*	*48.78*	*65.33*	*10.22*	*1.70*
31–40	**28.29**	**26.19**	**30.38**	**10.14**	**17.18**	**24.98**	**34.37**	**62.61**	**71.05**	**6.09**	**1.63**	*27.93*	*26.59*	*29.27*	*12.85*	*15.65*	*23.65*	*31.38*	*41.01*	*57.99*	*6.51*	*0.93*
41–50	**28.10**	**26.38**	**29.81**	**0.94**	**19.88**	**25.34**	**31.77**	**56.39**	**67.94**	**9.76**	**1.68**	*28.53*	*27.37*	*29.69*	*14.84*	*20.92*	*24.85*	*31.52*	*43.71*	*49.29*	*4.66*	*0.92*
51–60	**28.53**	**26.95**	**30.11**	**13.99**	**15.91**	**25.29**	**32.63**	**51.40**	**58.07**	**5.38**	**1.11**	*27.19*	*25.64*	*28.74*	*9.99*	*15.31*	*23.50*	*31.22*	*46.78*	*63.02*	*7.20*	*1.37*

All data are color-coded by sex (women = bold; men = italic). For each sex, four age groups (21–30,31–40, 41–50, 51–60) and all age groups are shown combined.

**Table 4:** Median, confidence interval (lower/upper limit), minimum (Min), percentile (P) 2.5, quartiles 1 and 3, percentile 97.5, maximum (Max), kurtosis and skewness of the pelvis parameters.

Age group	Median	Confidence Interval-Lower limit	Confidence Interval-Upper limit	Min	P 2.5	Quartile1	Quartile3	p 97.5	Max	Kurtosis	Skew-ness	Median	Confidence Interval-Lower limit	Confidence Interval-Upper limit	Min	P 2.5	Quartile1	Quartile3	p 97.5	Max	Kurtosis	Skew-ness
		Women											Men									
Pelvis distance (Spatial distance between PSIS left and PSIS right) (mm)																						
21–60	**97.96**	**96.54**	**99.38**	**41.73**	**71.86**	**88.61**	**107.67**	**128.28**	**155.08**	**4.23**	**0.24**	*98.11*	*96.11*	*100.10*	*60.20*	*70.54*	*88.81*	*112.68*	*148.54*	*174.12*	*3.37*	*0.81*
21–30	**99.96**	**97.56**	**102.36**	**41.73**	**76.37**	**91.93**	**108.01**	**122.95**	**127.79**	**6.20**	− **0.76**	*92.00*	*89.71*	*94.29*	*70.67*	*73.60*	*87.33*	*99.33*	*122.80*	*129.00*	*4.25*	*0.77*
31–40	**98.69**	**96.13**	**101.25**	**70.44**	**75.70**	**90.18**	**109.03**	**128.22**	**136.34**	**2.79**	**0.23**	*128.26*	*124.84*	*131.69*	*82.48*	*94.26*	*115.65*	*139.23*	*161.36*	*174.12*	*2.94*	− *0.07*
41–50	**102.35**	**98.86**	**105.84**	**68.18**	**73.01**	**90.12**	**109.82**	**148.64**	**155.08**	**3.79**	**0.70**	*93.00*	*90.79*	*95.20*	*61.47*	*72.52*	*85.25*	*102.26*	*113.93*	*125.09*	*3.03*	*0.04*
51–60	**94.57**	**91.91**	**97.23**	**61.47**	**65.12**	**83.24**	**102.29**	**115.01**	**116.81**	**2.41**	− **0.27**	*95.17*	*92.40*	*97.95*	*60.20*	*65.48*	*83.93*	*103.20*	*120.42*	*124.07*	*2.53*	− *0.21*
Pelvis hight (°) (Decline of the connecting line between PSIS left and PSIS right to the horizontal in the frontal plane in degrees)																						
21–60	− **0.42**	− **0.65**	− **0.19**	− **9.16**	− **3.93**	− **1.65**	**1.09**	**4.52**	**10.77**	**4.72**	**0.53**	− *0.42*	− *0.62*	− *0.21*	− *6.10*	− *4.26*	− *1.72*	*1.00*	*3.81*	*5.61*	*2.90*	*0.08*
21–30	**0.37**	− **0.12**	**0.85**	− **4.21**	− **3.73**	− **1.09**	**2.45**	**6.87**	**8.23**	**3.08**	**0.46**	− *1.00*	− *1.47*	− *0.53*	− *6.00*	− *4.20*	− *2.67*	*0.83*	*4.73*	*5.00*	*2.97*	*0.32*
31–40	− **0.67**	− **1.02**	− **0.32**	− **3.50**	− **3.44**	− **1.61**	**1.02**	**4.26**	**4.43**	**2.96**	**0.50**	− *0.60*	− *0.98*	− *0.22*	− *4.90*	− *3.86*	− *1.72*	*1.12*	*3.74*	*4.21*	*2.57*	*0.18*
41–50	− **1.44**	− **1.79**	− **1.08**	− **5.64**	− **4.20**	− **2.30**	− **0.02**	**2.72**	**3.47**	**2.94**	**0.29**	− *0.65*	− *1.03*	− *0.26*	− *5.29*	− *4.11*	− *1.46*	*0.68*	*3.64*	*5.61*	*3.58*	*0.34*
51–60	**0.04**	− **0.49**	**0.56**	− **9.16**	− **5.52**	− **1.37**	**1.15**	**4.38**	**10.77**	**6.26**	**0.17**	*0.16*	− *0.28*	*0.59*	− *6.10*	− *4.80*	− *1.47*	*1.63*	*3.81*	*4.12*	*2.81*	− *0.39*
Pelvis hight (mm) (Decline of the connecting line between PSIS left and PSIS right to the horizontal in the frontal plane in millimeter)																						
21–60	− **0.72**	− **1.10**	− **0.33**	− **12.36**	− **7.47**	− **2.87**	**1.85**	**8.43**	**15.40**	**4.04**	**0.54**	− *0.72*	− *1.08*	− *0.35*	− *9.69*	− *7.40*	− *3.07*	*1.74*	*6.50*	*10.51*	*2.83*	*0.11*
21–30	**0.70**	− **0.14**	**1.54**	− **7.61**	− **6.41**	− **1.92**	**4.57**	**11.81**	**13.81**	**3.07**	**0.43**	− *1.00*	− *1.76*	− *0.24*	− *9.00*	− *7.73*	− *4.00*	*1.33*	*6.53*	*8.00*	*2.72*	*0.21*
31–40	− **0.99**	− **1.61**	− **0.37**	− **6.68**	− **5.74**	− **2.78**	**1.68**	**7.59**	**8.58**	**3.26**	**0.59**	− *1.25*	− *2.08*	− *0.43*	− *9.46*	− *8.23*	− *3.58*	*2.72*	*7.21*	*10.51*	*2.60*	*0.25*
41–50	− **2.51**	− **3.15**	− **1.88**	− **8.54**	− **8.21**	− **3.91**	− **0.05**	**5.17**	**6.63**	**3.04**	**0.34**	− *1.02*	− *1.65*	− *0.38*	− *8.82*	− *6.99*	− *2.35*	*1.22*	*6.43*	*8.69*	*3.81*	*0.33*
51–60	**0.00**	− **0.82**	**0.81**	− **12.36**	− **9.68**	− **2.12**	**1.85**	**8.53**	**15.40**	**5.23**	**0.21**	*0.24*	− *0.46*	*0.94*	− *9.69*	− *6.93*	− *2.39*	*2.68*	*6.29*	*7.63*	*2.70*	− *0.26*
Pelvis torsion (PSIS L-PSIS R. twist around the transverse axis calculated from the mutual twisting of the surface normal on the two PSIS) (°)																						
21–60	**0.30**	− **0.21**	**0.81**	− **19.09**	− **8.53**	− **2.58**	**3.10**	**12.63**	**19.92**	**4.28**	**0.13**	*0.46*	− *0.10*	*1.02*	− *16.67*	− *11.17*	− *3.28*	*3.88*	*11.78*	*20.61*	*3.63*	− *0.02*
21–30	**0.32**	− **0.37**	**1.01**	− **9.96**	− **6.36**	− **1.91**	**2.35**	**7.38**	**13.39**	**4.31**	**0.26**	− *1.00*	− *2.11*	*0.11*	− *16.67*	− *12.33*	− *3.67*	*3.17*	*11.00*	*14.00*	*3.82*	− *0.01*
31–40	**0.83**	− **0.21**	**1.87**	− **19.09**	− **8.59**	− **2.24**	**4.25**	**10.35**	**15.81**	**3.90**	− **0.26**	*1.96*	*0.70*	*3.23*	− *15.99*	− *15.52*	− *2.44*	*4.58*	*13.66*	*20.61*	*3.95*	− *0.23*
41–50	**0.88**	− **0.39**	**2.15**	− **17.86**	− **9.80**	− **2.38**	**5.94**	**13.96**	**19.92**	**3.48**	**0.20**	*0.41*	− *0.66*	*1.48*	− *10.34*	− *8.56*	− *3.14*	*3.64*	*12.47*	*17.65*	*3.28*	*0.36*
51–60	− **1.23**	− **2.25**	− **0.21**	− **16.42**	− **10.69**	− **4.15**	**2.45**	**9.70**	**14.35**	**4.03**	− **0.04**	*0.54*	− *0.51*	*1.59*	− *13.89*	− *11.63*	− *3.46*	*3.89*	*9.48*	*11.58*	*2.77*	− *0.27*
Pelvis rotation (Rotation of the distance PSIS L – PSIS R in the transversal plane) (°)																						
21–60	**1.18**	**0.86**	**1.50**	− **7.78**	− **5.68**	− **1.10**	**3.54**	**7.33**	**10.73**	**2.82**	− **0.10**	− *0.16*	− *0.51*	*0.20*	− *12.40*	− *7.50*	− *2.87*	*2.30*	*5.87*	*14.02*	*3.37*	− *0.22*
21–30	**2.20**	**1.63**	**2.76**	− **6.79**	− **5.48**	**0.62**	**3.83**	**6.95**	**8.89**	**3.63**	− **0.66**	− *0.83*	− *1.57*	− *0.09*	− *10.33*	− *7.47*	− *3.33*	*2.00*	*5.80*	*7.00*	*2.59*	− *0.18*
31–40	**0.61**	**0.00**	**1.21**	− **5.25**	− **4.36**	− **1.28**	**3.24**	**7.18**	**9.22**	**2.44**	**0.21**	*0.34*	− *0.40*	*1.08*	− *8.52*	− *7.01*	− *2.13*	*3.03*	*6.16*	*14.02*	*3.88*	*0.16*
41–50	**0.67**	− **0.10**	**1.43**	− **7.78**	− **7.44**	− **1.78**	**3.80**	**7.43**	**10.73**	**2.70**	− **0.10**	− *0.15*	− *0.76*	*0.47*	− *8.36*	− *6.58*	− *2.47*	*2.63*	*5.55*	*6.76*	*2.51*	− *0.15*
51–60	**0.50**	− **0.15**	**1.15**	− **5.69**	− **4.91**	− **1.53**	**2.92**	**7.75**	**9.89**	**2.92**	**0.33**	− *0.43*	− *1.20*	*0.35*	− *12.40*	− *10.30*	− *3.59*	*1.91*	*5.34*	*5.80*	*3.14*	− *0.56*

All data are color-coded by sex (women = Bold; men = italic). For each sex, four age groups (21–30, 31–40, 41–50,51–60) and all age groups are shown combined.

The formulae for calculating each evaluation parameter have been previously published by Ohlendorf et al.^6^.

Figure 1 shows the back scan and the six markers set on the bare back including their names. All abbreviations are explained at the bottom of the figure.

### Statistical analysis

The first step required was to check for the normal distribution of the data by means of the Kolmogoroff–Smirnoff–Lilliefors test. Accordingly, parametric or non-parametric tests were used. Descriptive parameters for the individual variables were calculated and the general distribution was characterized based on the minimum, 2.5, 25, 50, 75, 97.5 percentiles and the maximum, while the kurtosis and skewness of the distribution were also determined and the tolerance range (TR) with its lower (loL) and upper limits (uL) and the confidence interval (CI) with the left (leL) and right limits (rL)^46,47^. With respect to the overall descriptive data presented here, the 2.5 and 97.5 percentiles defined the normal range of the data as we had utilized the 95% CI and 95% TR.

The upper and lower limits for 95% of all values (= ± 2σ values) defined the tolerance regions. Thus, the data that was found in about 95% of the examined subjects was considered as normal. Consequently, the tolerance range estimated the central part of the 95% of the value of the measured subject population. In contrast, the two-sided 95% CI indicated the possible range for the mean or median values, depending on the distribution quality.

Differences between the gender and age groups were tested via an ANOVA. In order to utilize non-normally distributed values, prior to the ANOVA, a normal rank transformation was applied. If significance in the ANOVA was found, a post hoc test was applied^48^.

Dependencies between the variables were tested by means of regression analysis between the individual variable and the age and body mass index within the gender groups.

A factor analysis was applied to the two gender groups to identify common factors within the measured variables. The statistical analysis was carried out using Matlab (Version 2020a). The significance level was set to α = 0.05.

### Ethics approval and consent to participate

The study was in accordance with the 1964 Helsinki Declaration and its later amendments and was approved by the local medical ethics committee of the Faculty of Medical Science, Goethe University Frankfurt, Germany (Approval No. 303/16). No patients were involved. All volunteers were healthy and informed about the study design before giving written informed consent.

## Results

A total number of 800 (407f/393 m) subjects participated in this study (Table 1). The number of subjects in the 4 age groups ranged between 92 (male, 21 to 30 years) and 106 (female, 21 to 30 years). The activity questionnaire reveald that the inactivity of the present sample (35%) is close to the range of the general German population with 41%^49^.

### Descriptive data

Table 2 contains the median, confidence interval (lower/upper limit), minimum (Min.), percentile (P) 2.5, quartiles 1 and 3, percentile 97.5, maximum (Max.), kurtosis and skewness of the spine parameters for women (= red) and men (= blue). Table 3 contains the same information for the shoulder parameters and Table 4 for the pelvis parameters. A test on the normal distribution revealed that a few of the subgroups were not normally distributed. This can also be seen when looking at the kurtosis and skewness parameters. However, for the majority of the variables, the kurtosis was around 3 for most age and gender subgroups, while the skewness was around 0.

A description of the upper body posture for each sex, summarized from the results of the four age groups (21–30, 31–40, 41–50 and 51–60 years), is given below.

Tables 2, 3, 4.

#### Women

The median posture of all women, regardless of age, was determined as follows:

The trunk length S was 42 mm longer than the trunk length D, with the trunk marginally tilted to the left (frontal trunk decline and axis decline) and about 3° anteriorly. In contrast, the root mean squared deviation of the median line of the distance C7—Center of the PSIS marker showed a rightward deviation of 3.84°. The thoracic kyphosis angle was approximately 6° greater than the lumbar lordosis angle, whereas the thoracic bending angle was only marginally greater than the lumbar bending angle (13.90° and 14.25°, respectively). The spinous processes of the spine showed a right torsion of about 4.18°.

In relation to the shoulder region, the left scapula was about 2 mm more caudal (scapula height and angle), whereas the right scapula was about 1.75° more anteriorly rotated (scapula rotation). The pelvis was basically balanced and only marginally rotated to the right anteriorly (pelvis rotation of 1.18°).

The deviations from this median posture across all age groups in the individual decades were not very pronounced. With regard to the trunk length and scapula distance, differences of ± 10 and 13 mm, respectively, were observed, whilst with regard to pelvis distance, differences of ± 3 mm were noted. All other spine, shoulder and pelvis parameters deviated by a maximum of ± 2°. Only the kyphosis angle had a margin of maximum ± 4°.

#### Men

The median posture of all the men was similar to that of the female participants. Only the difference between the trunk length S and trunk length D which was approximately 45 mm, was approximately 3 mm greater than that of the women. Furthermore, the trunk length D for the men was 35 mm longer than for the women, thus explaining 26% of the height difference between the men and women (1669 mm for the women and 1804 mm for the men). The male scapula distance was also approximately 27 mm larger. In addition, the thoracic kyphosis angle was about 5° less, the lumbar lordosis angle about 17° less, and the lumbar bending angle was about 3° smaller than those of the women. Only the thoracic bending angle was 2° greater than that of the females.

The deviations from this median posture across all age groups in the individual decades were not very pronounced. With regard to the scapula distance, the trunk length and the pelvis distance, differences of ± 11 and 12 mm, respectively, and + 30/− 6 mm could be observed. All other spine, shoulder and pelvis parameters differed by a maximum of ± 2°. Only the thoracic kyphosis angle had a margin of maximum ± 4°.

### Factor age and sex

Sex-dependent and sex and age differences were found in 11 and 12 variables, respectively (Table 5). The female subgroup showed a significant dependency with age for 9 variables, while the male subgroup showed a significant dependency for 7 variables.

**Table 5:** Sex and age specific differences based on the predefined groups.

	Difference between gender			Difference between gender and age			Post hoc–comparison
	t	*p*	df	F	*p*	df	
Trunk length S	− 18.35	** < 0.001**	797	63.5	** < 0.001**	7.791	f3,f4 < f1 < m1,m2,m3,m4 ; f3,f4 < f2 < m1,m2,m3,m4; m1,m2 < m3
Kyphosis angle	6.50	** < 0.001**	797	15.4	** < 0.001**	7.791	m1 < f1,m2,m3 < f1,f3,f4; m1 < m4 < f4
Lordosis angle	19.72	** < 0.001**	797	59.0	** < 0.001**	7.791	m1,m2,m3,m4 < f1,f2,f3,f4
Lumbar bending angle	15.53	** < 0.001**	797	37.7	** < 0.001**	7.791	m1,m2,m3,m4 < f1 < f2; m1,m2,m3,m4 < f3,f4
Thoracic bending angle	− 8.36	** < 0.001**	797	11.7	** < 0.001**	7.791	f1,f2,f3 < m1,m2,m3,m4; f4 < m1,m4
Abs SD of rotation	3.76	** < 0.001**	797	4.5	** < 0.001**	7.791	f4,m1,m2,m3 < f1
Abs scapular rotation	3.32	** < 0.001**	797	3.7	** < 0.001**	7.791	m1,m2,m3 < f1
Abs pelvis rotation	0.25	0.799	797	1.0	0.431	7.791	
Abs SD of lateral deviation	− 0.18	0.861	797	3.3	**0.002**	7.791	f4 < f2,m2; f2,m3 < m2
Scapular distance	− 15.97	** < 0.001**	797	57.9	** < 0.001**	7.791	f1 < f4 < f3,m1,m2,m3,m4; f2 < f3,m1,m2,m3,m4; f3,m1 < m3,m4; m2 < m4
Scapular angle right	2.47	**0.014**	797	2.0	0.053	7.791	
Scapular angle left	2.95	**0.003**	797	2.2	**0.033**	7.791	m4 < f1
Abs sagittal trunk decline	1.00	0.318	797	1.3	0.265	7.791	
Abs axis decline	− 0.83	0.406	797	0.4	0.912	7.791	
Abs pelvis height	0.96	0.339	797	1.1	0.338	7.791	
Abs frontal trunk decline	0.79	0.430	797	1.4	0.183	7.791	
Abs pelvis torsion	− 2.12	**0.034**	797	3.2	**0.002**	7.791	f1 < f3,m2,m4
Abs scapular height	1.26	0.209	797	4.4	** < 0.001**	7.791	f2, f4, m3 < f1; m3 < m2

The post hoc comparison shows differences between female (f) and male (m) subjects according to the four age groups: 1: 21–30 years; 2: 31–40; 3: 41–50; 4: 51–60. If Abs is placed in front of a parameter, only the absolute values are integrated into the calculations, i.e. only the extent without the direction of movement. Significant variables are highlighted in bold.

**Figure 2:**
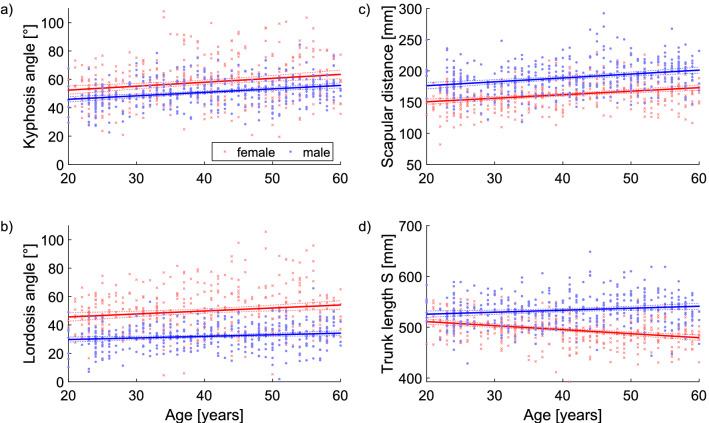
Age-dependent differences for the kyphosis and lordosis angles, and for the scapular distance and trunk length.

The thoracic kyphosis angle and the lumbar lordosis angle were found to be greater in females than in males and increased with age independently of sex. The scapular distance was found to be smaller for females and also increased with age independently of sex. The trunk length was smaller for female subjects, in general, but increased for male subjects and decreased for female subjects with increasing age (Table 6, Fig. 2).

**Table 6:** Age dependent regression analysis.

VariableName	Slope [lower .. Upper]	
	Female	Male
Trunk length S	− **0.79 [**− **1.02..**− **0.56]**	**0.39 [0.13..0.66]**
Kyphosis angle	**0.28 [0.15..0.41]**	**0.24 [0.16..0.33]**
Lordosis angle	**0.21 [0.08..0.34]**	**0.11 [0.04..0.19]**
Lumbar bending angle	0.03 [− 0.01..0.06]	**0.03 [0.00..0.05]**
Thoracic bending angle	0.02 [− 0.02..0.05]	**0.04 [0.01..0.08]**
Abs SD of rotation	− **0.03 [**− **0.05..**− **0.01]**	0.01 [− 0.01..0.03]
Abs scapular rotation	− **0.02 [**− **0.04..0.00]**	0.01 [− 0.01..0.03]
Abs pelvis rotation	0.00 [− 0.02..0.02]	0.00 [− 0.02..0.02]
Abs SD of lateral deviation	0.00 [− 0.02..0.01]	− 0.01 [− 0.03..0.01]
Scapular distance	**0.57 [0.38..0.75]**	**0.62 [0.41..0.84]**
Scapular angle right	− **0.10 [**− **0.18..**− **0.01]**	− 0.03 [− 0.09..0.04]
Scapular angle left	− 0.02 [− 0.11..0.08]	− 0.03 [− 0.09..0.03]
Abs sagittal trunk decline	0.02 [0.00..0.04]	0.01 [− 0.02..0.02]
Abs axis decline	0.00 [− 0.01..0.02]	0.00 [− 0.01..0.02]
Abs pelvis height	− 0.01 [− 0.02..0.01]	− 0.01 [− 0.02..0.01]
Abs frontal trunk decline	0.00 [− 0.01..0.01]	**0.01 [0.00..0.02]**
Abs pelvis torsion	**0.04 [0.01..0.07]**	0.00 [− 0.04..0.03]
Abs scapular height	− **0.08 [**− **0.13..**− **0.04]**	− 0.01 [− 0.05..0.03]

Slope of the age dependent relationship with the 95% confidence interval of the slope [lower .. upper]. If Abs is placed in front of a parameter, only the absolute values are integrated into the calculations, i.e. only the extent without the direction of movement. Significant variables are highlighted in bold.

### Factor BMI

**Figure 3:**
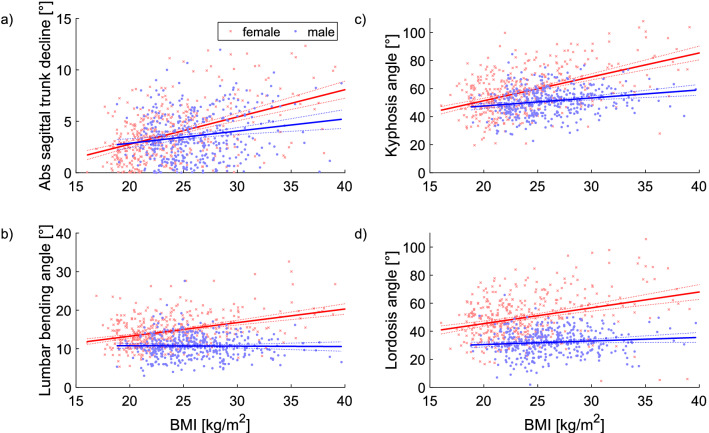
The BMI-dependent relationships of selected variables. *Legend* Abs, Absolute value.

For both sexes, the thoracic kyphosis and lumbar lordosis angles, the scapula distance and the absolute sagittal trunk decline increased with the BMI. The dependency was greater for the female subjects than for the male subjects. The trunk length, lumbar bending angle and the thoracic bending angle, absolute pelvis torsion and the left scapula angle increased with the BMI for the female cohort. The scapular angle to the right decreased with increasing BMI for the male subjects. These data are presented in detail in Table 7. In addition, selected regression analyses are illustrated in Fig. 3.

**Table 7:** BMI dependent regression analysis.

	Female	Male
Trunk length S	− **1,27 [**− **1,86..**− **0,68]**	− 0,14 [− 1,01..0,73]
Kyphosis angle	**1,70 [1,41..2,00]**	**0,57 [0,31..0,84]**
Lordosis angle	**1,13 [0,81..1,44]**	**0,25 [0,01..0,50]**
Lumbar bending angle	**0,35 [0,27..0,43]**	− 0,01 [− 0,10..0,08]
Thoracic bending angle	**0,10 [0,01..0,19]**	− 0,02 [− 0,13..0,09]
Abs SD of rotation	− 0,03 [− 0,08..0,02]	0,01 [− 0,06..0,07]
Abs scapular rotation	0,01 [− 0,04..0,05]	− 0,01 [− 0,07..0,05]
Abs pelvis rotation	0,02 [− 0,02..0,06]	− 0,01 [− 0,07..0,06]
Abs SD of lateral deviation	0,04 [0,00..0,08]	0,01 [− 0,06..0,07]
Scapular distance	**2,14 [1,70..2,58]**	**2,33 [1,64..3,03]**
Scapular angle right	− 0,22 [− 0,44..0,00]	− **0,27 [**− **0,47..**− **0,08]**
Scapular angle left	− **0,40 [**− **0,64..**− **0,16]**	− 0,16 [− 0,36..0,04]
Abs sagittal trunk decline	**0,27 [0,22..0,31]**	**0,12 [0,06..0,18]**
Abs axis decline	− 0,02 [− 0,05..0,02]	0,00 [− 0,04..0,04]
Abs pelvis height	− 0,02 [− 0,05..0,01]	− 0,01 [− 0,05..0,03]
Abs frontal trunk decline	0,01 [− 0,01..0,03]	0,02 [− 0,01..0,04]
Abs pelvis torsion	**0,11 [0,04..0,18]**	0,10 [0,00..0,20]
Abs scapular height	− 0,04 [− 0,15..0,08]	− 0,02 [− 0,16..0,12]

Slope of the BMI dependent relationship with the 95% confidence interval of the slope [lower … upper]. If Abs is placed in front of a parameter, only the absolute values are integrated into the calculations, i.e. only the extent without the direction of movement. Significant variables are highlighted in bold.

### Factor BMI and age

**Figure 4:**
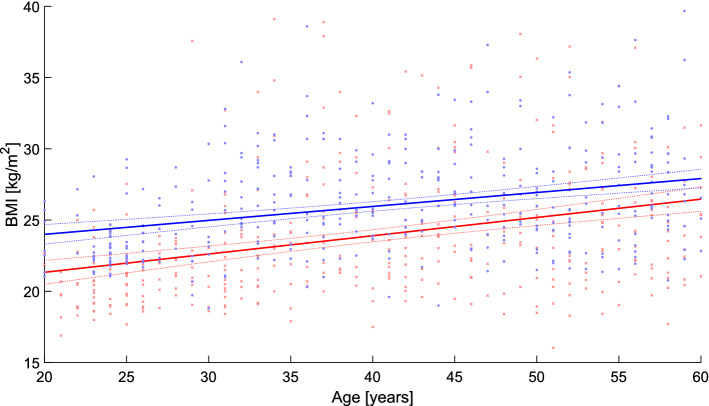
Relationship between the BMI and age.

Figure 3 shows the correlation per sex between age and the BMI. In both sexes, an increase in BMI with increasing age can be seen (Fig. 4).

### Factor analysis

**Figure 5:**
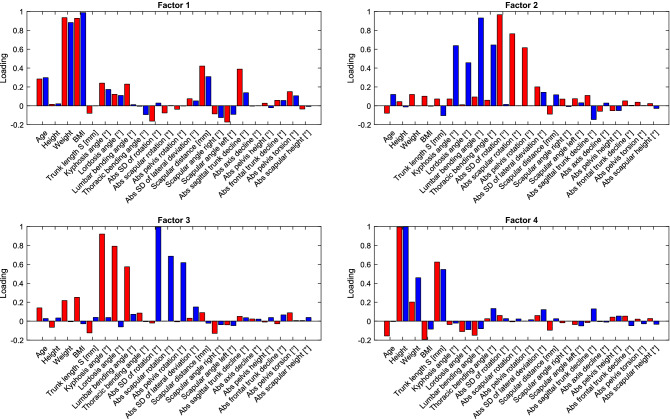
Factor loadings of the first 4 factors. The gender-specific factors were calculated for the two gender groups (red: female; blue: male). *Legend* Abs, Absolute value.

Figure 5 depicts the gender-dependent factor loadings. The first factor loads on the variables of weight and BMI, with a smaller loading on age (the correlation with age is shown in Fig. 2), the scapular distance and the absolute sagittal trunk decline. The second and third factors are different between the genders. For the second factor, the male subjects varied more in the spine angles (kyphosis angle, lordosis angle, lumbar bending angle and thoracic bending angle), while the female subjects varied more in the rotation of the scapular and the pelvis. The third factor showed a similar relationship as the 2nd factor, but with the opposite gender. The 4th factor loads on the height-dependent variables such as height and trunk length.

## Discussion

This study generated standard values for the dorsal upper body statics of men and women of working age (21–60 years) and analyzed whether gender, age or BMI were influencing factors. The method of video rasterstereography was used to collect the data in order to faithfully represent the dorsal surface of the trunk in three dimensions which is dependent on the muscle and fat of the bare back^5,7,27–31,45,50^. In comparison with other BMI data of men and women of equivalent age groups recorded in Germany, the present BMI mean values in each age and sex group correspond to these previously published data^51–53^, with the BMI increasing with age—both in the previous data and in the present findings. We, therefore, assume that the sample drawn in the present study represents the general German population, indicated by the similar values for the BMI, body height and weight. Thus, it is possible to draw resonable conclusions about the population from this sample of the present study.

The medians of the parameters show a rather balanced upper body statics across all the age groups in the women which is dependent on the body height, or the slightly increasing BMI with age (trunk length, scapula distance and pelvis distance). All other parameters of the spine, shoulder and pelvis region deviate by a maximum of ± 2°, except the thoracic kyphosis angle with a margin of maximum ± 4°. The statics of the males are similar to those of the females across all age groups when taking into account the constitutional parameters (trunk length, scapula distance and pelvis distance). In general, the female thoracic kyphosis angle is about 5° greater than that of men, while the female lumbar lordosis angle is about 17° greater and the female lumbar bending angle about 3° greater than in males. However, the thoracic bending angle was found to be approximately 2° smaller in men. The scapula and pelvis distance as well as the trunk length were determined by constitutional conditions in both sexes.

Overall, the data are quite similar to a normal distribution, with a kurtosis around 3 and a skewness around 0. In a very limited number of subgroups, the data were significantly different to a normal distribution which is why all the data were rank transformed prior to any further analysis.

With increasing age, the thoracic kyphosis and lumbar lordosis angles (♀ > ♂) and scapular distance (♂ > ♀) increased regardless of sex. The trunk length was generally shorter in the female subjects but increased in the male subjects, while it decreased with age in the female subjects. Within each sex, most parameters of the spine, shoulder and pelvis differed by a maximum of ± 2°, with the exception of the kyphosis angle, which had a range of a maximum of ± 4°.

Since the BMI was observed to increase slightly with age (Fig. 4), this was found to be as a significant influencing factor. Thus, in both sexes, the thoracic kyphosis and lumbar lordosis angles, the scapula distance and the absolute sagittal trunk decline also increased with the BMI, although this dependence was more pronounced in the female sex. In addition, in the females, the torso length, lumbar flexion angle and thoracic flexion angle, absolute pelvic torsion and left scapula angle increased with BMI. The pelvic position was to be related to age, sex or BMI.

These descriptive differences can also be confirmed with factor analysis. The first and, thus, dominant factor explained the relationship between weight (as well as BMI) and shoulder distance, absolute value sagittal trunk tilt, and to a lesser extent the relationship between weight and the kyphosis angle, lordosis angle and lumbar flexion angle. The second and third factors (sagittal and transverse planes, respectively) influenced the sagittal angles (kyphosis angle, lordosis angle, lumbar flexion angle and thoracic flexion angle) and transverse rotations (absolute value SD of rotation, absolute value scapular rotation and absolute value pelvic rotation). In summary, there was a good relationship within the levels and a poor relationship between the two levels. The male subgroup had higher variability in the sagittal angles, while the female subgroup had higher variability in the rotation angles. Only the fourth factor showed the obvious relationship between body size and trunk length.

These results concerning the kyphosis and lordosis angles and the sagittal trunk inclination in relation to sex, age and BMI concur with those determined by Celan et al.^54^ in 250 subjects between 20 and 70 years of age. Drzał-Grabiec et al.^16^ addressed the same question in two studies; this group also used video rasterstereography while consulting similar evaluation parameters. Their comparison of 70 older subjects (60–90 years) with 70 younger subjects (20–25 years) showed a significant difference in each evaluation parameter. Similar results were shown by gender comparisons of both age groups: an increasing trunk tilt and thoracic kyphosis were the most significant age-related parameters (♀ > ♂). Furthermore, a gender comparison over 60 years showed a significantly higher angle of lumbar lordosis in men. The increase in kypohosis with age in women was also confirmed by Sigh et al.^55^ using electromagnetic tracking.

Zapallá et al.^56^ concluded in their systematic review that kyphosis increases with age and varies significantly below 40 years and above 60 years, but not by gender^56^. Single studies could also confirm the correlation between the increase of thoracic kyphosis with age. The accentuation of thoracic kyphosis with age is observed in both women and men and is due to multifactorial genesis. Since bone and muscle also interact closely chemically and metabolically, fat infiltration is also observed in age-related bone and muscle loss (sarcopenia)^57^. Furthermore, there is an age-related degenerative reduction in mitochondrial function as well as a reduction in the degree of interconnectedness of neurons at the neuronal level, which is also associated with a decrease in responsiveness as well as a reduction in processing speed with regard to motor function among other things^58–61^. Equivalent age-related changes in lumbar lodosis have not been demonstrated^55,62^. However, Roussouly et al.^63^ concluded interindividual reciprocal relationships between the sacral alignment, sacral tilt, pelvic dip and lumbar lordosis characteristics. Here, the lordotic curvature, tilt angle of lordosis, position of the apex and the number of lordotic vertebrae are determined by the angle of the upper endplate of S1 with respect to the horizontal axis.

Gender differences in kyphosis can also be observed: women are more affected by age-related kyphosis than men. Postmenopausal women have a higher predisposition to develop osteoporosis and, thus, a higher risk of bone fractures^57^ and, furthermore, in this context, the strength of the extensors of the back muscles is in inverse proportion to the (hyper)kyphosis^64^. Moreover, genoid obesity is more common in women which affects the center of mass compared to male abdominal obesity and, thus, they may also differ in their sagittal body posture^15,65^. With respect to the BMI, increased weight may be causative of the increased mechanical loading of the lumbar spine with a resulting increase in the lordotic angle^66^. It should also be noted that breast size correlates positively with BMI but not with age^18^.

While parameters of the frontal plane provide information about symmetry or asymmetry, such as a shoulder or pelvic obliquity or a scoliotic malposition, the shock absorber function of the superimposed vertebrae can be deduced from the sagittal view based on the expression of the thoracic kyphosis or lumbar lordosis and related expressions (flat back, hyperkyphosis, hyperlordosis). From a biomechanical point of view, deviations from the axis of symmetry can lead to muscular discomfort due to imbalances while sagittal deviations from the optimal shock absorber function can lead to functional or structural pathologies. As a consequence, pain and/or degeneration of the intervertebral disc or facet joints, for example, may result due to non-physiological spinal function or biomechanics^67,68^.

Understanding the patterns of variation in the upper body posture in all three levels of men and women, both gender-specific and gender-comparative, as well as age-related potential changes in expression, may potentially help to identify degenerative changes in diseases, such as Scheuermann's or ankylosing spondylitis. These observable changes may not be confined to only to the spine, but their effects may also be observed in the shoulder and pelvic regions and thus, may enable a better understanding of their interaction and, in the best case, to detect them at an early stage which would be beneficial to the patient's health. Furthermore, the available data may help to detect predictors for incidental surgeries in the trunk, possibly at an early stage, since it is recorded as a whole. Until now, studies^19–21,24^ using similar measurement methods in the literature have mainly focused on spinal parameters, in particular the kyphosis and lordosis angles or sagittal torso inclination, with the shoulder and pelvic regions receiving only rudimentary attention.

In addition to the clinical issues, a prevailing interest also lies with regard to the financial aspects, especially under the perspective of constantly rising health care costs in industrialized nations. Insurance companies and healthcare institutions are under increasing pressure to establish objective evaluations of impairments related to illness or injury, e.g., spinal disorders or back pain. Furthermore, surgery can also alter the upper body posture.

Soft tissue artifacts must be taken into account in the data analysis because video rasterstereography captures the trunk surface in three dimensions and, consequently, the dorsal upper body posture displayed in the software is a construct of anatomically visible structures and soft tissue structures (adipose tissue and musculature), which is subject to biological variability and this, therefore, sometimes makes it difficult to identify correctly the given anatomical landmarks as landmarks during the measurements. However, a trained examiner can easily identify them, even with a patient of high body weight. Furthermore, it must be considered here that the data of the upper body posture comprise only indirect information about the 3-dimensional shape of the spine or all the back parameters, because their generation is achieved via light projections. Thus, in order to adjust postural fluctuations due to respiration, heartbeat or imperfect proprioception into account in the data and to reduce them mathematically, on the one hand, a back image is taken at 50 frames/second and, on the other hand, three images are statistically averaged. When comparing the existing images with X-ray images, it must be taken into account that the angles in the X-ray images are determined according to anatomical criteria, whereas geometrical criteria are taken into account in video rasterstereography. Therefore, although video rasterstereography cannot be used in a direct comparison with radiographs, it can be used as a non-invasive method as an alternative for intermediate diagnosis. Porto and Okazaki^69^ in their review confirmed the differences in the data interpretation of images of dorsal statics that were originating from X-ray and photogammetry due to their discrepancies in the procedures and angle calculations.

Furthermore, the interpretation of a large number of objective evaluation parameters when classifying into normal/abnormal posture is very often based on subjective empirical values; a sufficient number of standard values have been lacking to such an extent up to the present day. In the future, further mathematical methods, such as data mining or machine learning techniques, could support the better extraction of medically relevant information from available data sets. In addition, data analysis should take into account if the influence of handedness on the expression of back geometry is feasible. In the context of this study, this aspect is rather negligible since approximately 95% of the study participants were right-handed. A more detailed investigation of exclusively left-handed subjects would be desirable in future studies.

Overall, no conspicuous, age-related signs of deterioration in posture were observed in the healthy adults aged 21–60 years presented here. Accordingly, further analyses should focus, in particular, on persons aged 60 years and over with the question of finding out at which age body posture-related changes of the spine occur most intensely and with which parameters these changes can be proven. In this context, Yukawa et al.^70^ were only able to demonstrate remarkable age- and sex-specific differences in the sagittal alignment of the spine and pelvis in asymptomatic individuals from the 7th decade of life and beyond. From this age on, among other things, an increasing hyperkyphosis becomes apparent which is not only a cosmetic deformity, but is also associated with an increased risk of various health impairments such as poor physical constitution, lung problems, falls or fractures. Since kyphosis is more pronounced in women than in men and negative kyphosis-related health problems are known, segmental training against thoracic kyphosis is recommended in old age, as well as total body training against the background of sorcopenia, in order to maintain, sustainably, the entire aging human body. However, it must be taken into account that the alignment of the human spine can vary greatly between individuals, and that this can also be attributed to a multicausal genesis among other factors^63^. Here, work-related influences or everyday, or sporting, activities may have more influence on the upper body constitution with increasing years of work than age or gender. The recording of these further influencing parameters was not possible within the framework of this research project, but should be taken into account in future analyses on the basis of the available data, as should the aspect of changes over time. Another aspect to consider is pregnancy in women. Here it has been proven that various aspects of biomechanics can occur and back pain can develop, although these do not correlate with spinal changes^71^. Furthermore, Betsch et al.^38^ also proved by means of raster stereography that reversible increases in kyphosis and lodosis occur during pregnancy. This result should therefore be related to the normal values in future analyses.

Considering the above mentioned limitations, innovative methods, especially non-ionizing technologies such as the back scanner, should be further developed so that the 3D posture and morphology of the spine may be ideally used for the purpose of clinical assessment in physical medicine and rehabilitation, of course by using the existing standard/reference values presented in this study.

## Conclusions

The present data, recorded by video rasterstereography, could serve as useful guidelines for the prediction of normal dorsal upper body posture of subjectively healthy Caucasian (european) men and women of between 20 and 60 years of age. In general, a balanced, symmetrical posture is present in both sexes and in each age group, subject to constitutional factors (sex, height, weight and BMI). Most noticeably, women have greater lordosis and kyphosis angles as well as the lumbar bending angle in this regard. The distance between the shoulder blades is more pronounced in men. These parameters are also age-dependent and increase with age, as does the BMI. Pelvic parameters are independent of age and sex. Thus, for example, the disease progression of sagittal plane deformities, such as Scheuermann's disease, could be monitored.

## Data Availability

All data generated or analysed during this study are included in this published article.

## Abbreviations

BMI: Body mass index
SD: Standard deviation
Min: Minimum
Max: Maximum
P: Percentile
TR: Tolerance range
CI: Confidence interval
loL: Lower limit
uL: Upper limit
leL: Left limit
rL: Right limit

## References

[1] Abubaker AO, Raslan WF, Sotereanos GC (1993). Estrogen and progesterone receptors in temporomandibular joint discs of symptomatic and asymptomatic persons: A preliminary study. J. Oral Maxillofac. Surg..

[2] Bush FM, Harkins SW, Harrington WG, Price DD (1993). Analysis of gender effects on pain perception and symptom presentation in temporomandibular pain. Pain.

[3] Conti PC, Ferreira PM, Pegoraro LF, Conti JV, Salvador MC (1996). A cross-sectional study of prevalence and etiology of signs and symptoms of temporomandibular disorders in high school and university students. J. Orofac. Pain.

[4] Farenc I, Rougier P, Berger L (2003). The influence of gender and body characteristics on upright stance. Ann. Hum. Biol..

[5] Ohlendorf D, Adjami F, Scharnweber B, Schulze J, Ackermann H, Oremek GM (2018). Standard values of the upper body posture in male adults. Adv. Clin. Exp. Med..

[6] Ohlendorf D, Gerez A, Porsch L, Holzgreve F, Maltry L, Ackermann H (2020). Standard reference values of the upper body posture in healthy male adults aged between 41 and 50 years in Germany. Sci. Rep..

[7] Ohlendorf D, Fisch V, Doerry C, Schamberger S, Oremek G, Ackermann H (2018). Standard reference values of the upper body posture in healthy young female adults in Germany: An observational study. BMJ Open.

[8] Ohlendorf D, Sosnov P, Keller J, Wanke EM, Oremek G, Ackermann H (2021). Standard reference values of the upper body posture in healthy middle-aged female adults in Germany. Sci. Rep..

[9] Ohlendorf D, Kaya U, Goecke J, Oremek G, Ackermann H, Groneberg DA (2021). Standard reference values of the upper body posture in healthy male adults aged between 31 and 40 years in Germany-an observational study. J. Physiol. Anthropol..

[10] Ohlendorf D, Krüger D, Christian W, Ackermann H, Keil F, Oremek G (2022). Standard reference values of the upper body posture in healthy male adults aged between 51 and 60 years in Germany. Sci. Rep..

[11] Faller, A., Schünke, M. Der Körper des Menschen: Einführung in Bau und Funktion: Thieme (2016).

[12] Schiebler TH, Korf H-W (2007). Anatomie: Histologie, Entwicklungsgeschichte, makroskopische und mikroskopische Anatomie, Topographie.

[13] Medizin, R. L. Dtsch Arztebl International. 100(50):3306 (2003).

[14] Abrisham SMJ, Ardekani MRS, Mzarch MAB (2020). Evaluation of the normal range of thoracic kyphosis and lumbar lordosis angles using EOS imaging. Maedica.

[15] Fon GT, Pitt MJ, Thies AC (1980). Thoracic kyphosis: Range in normal subjects. AJR Am. J. Roentgenol..

[16] Tunckale T, Gurdal SO, Caliskan T, Topcu B, Yuksel MO (2021). The impact of various breast sizes of women on vertebral column and spinopelvic parameters. Turk. Neurosurg..

[17] Karaaslan O, Demirkiran HG, Silistreli O, Sonmez E, Bedir YK, Can M (2013). The effect of reduction mammaplasty on the vertebral column: A radiologic study. Sci. World J..

[18] Coltman CE, Steele JR, McGhee DE (2017). Breast volume is affected by body mass index but not age. Ergonomics.

[19] Drzał-Grabiec J, Rykała J, Podgórska J, Snela S (2012). Changes in body posture of women and men over 60 years of age. Ortop. Traumatol. Rehabil..

[20] Iorio J, Lafage V, Lafage R, Henry JK, Stein D, Lenke LG (2018). The effect of aging on cervical parameters in a normative north american population. Glob. Spine J..

[21] Park MS, Moon SH, Lee HM, Kim SW, Kim TH, Lee SY (2013). The effect of age on cervical sagittal alignment: Normative data on 100 asymptomatic subjects. Spine.

[22] Brandes R, Lang F, Schmidt RF (2019). Physiologie des Menschen.

[23] Corpas Cobisa E, Vicent D, Granizo V, Sanz E, Ruiz TA (1989). Changes with age in adiposity and lean body mass in a healthy Spanish population. Nutr. Hosp..

[24] Drzał-Grabiec J, Snela S, Rykała J, Podgórska J, Banaś A (2013). Changes in the body posture of women occurring with age. BMC Geriatr..

[25] Pavlova AV, Muthuri SG, Cooper R, Saunders FR, Gregory JS, Barr RJ (2018). Body mass index and waist circumference in early adulthood are associated with thoracolumbar spine shape at age 60–64: The medical research council national survey of health and development. PLoS ONE.

[26] Don R, Capodaglio P, Cimolin V, Benedetti MG, D'Osualdo F, Frigo C (2012). Instrumental measures of spinal function: Is it worth? A state-of-the art from a clinical perspective. Eur. J. Phys. Rehabil. Med..

[27] Betsch M, Rapp W, Przibylla A, Jungbluth P, Hakimi M, Schneppendahl J (2013). Determination of the amount of leg length inequality that alters spinal posture in healthy subjects using rasterstereography. Eur. Spine J..

[28] Drerup B (2014). Rasterstereographic measurement of scoliotic deformity. Scoliosis.

[29] Betsch M, Wild M, Rath B, Tingart M, Schulze A, Quack V (2015). Radiation-free diagnosis of scoliosis : An overview of the surface and spine topography. Der Orthopade..

[30] Wild M, Kuhlmann B, Stauffenberg A, Jungbluth P, Hakimi M, Rapp W (2014). Does age affect the response of pelvis and spine to simulated leg length discrepancies? A rasterstereographic pilot study. Eur. Spine J..

[31] Mohokum M, Mendoza S, Udo W, Sitter H, Paletta JR, Skwara A (2010). Reproducibility of rasterstereography for kyphotic and lordotic angles, trunk length, and trunk inclination: A reliability study. Spine.

[32] Schulein S, Mendoza S, Malzkorn R, Harms J, Skwara A (2013). Rasterstereographic evaluation of interobserver and intraobserver reliability in postsurgical adolescent idiopathic scoliosis patients. J. Spinal Disord. Tech..

[33] Mohokum M, Schulein S, Skwara A (2015). The validity of rasterstereography: A systematic review. Orthopedic reviews..

[34] Guidetti L, Bonavolonta V, Tito A, Reis VM, Gallotta MC, Baldari C (2013). Intra- and interday reliability of spine rasterstereography. Biomed. Res. Int..

[35] Schroeder J, Reer R, Braumann KM (2015). Video raster stereography back shape reconstruction: A reliability study for sagittal, frontal, and transversal plane parameters. Eur. Spine J..

[36] Weiss HR, Elobeidi N (2008). Comparison of the kyphosis angle evaluated by video rasterstereography (VRS) with x-ray measurements. Stud. Health Technol. Inform..

[37] Colombo T, Mangone M, Agostini F, Bernetti A, Paoloni M, Santilli V (2021). Supervised and unsupervised learning to classify scoliosis and healthy subjects based on non-invasive rasterstereography analysis. PLoS ONE.

[38] Betsch M, Wehrle R, Dor L, Rapp W, Jungbluth P, Hakimi M (2015). Spinal posture and pelvic position during pregnancy: A prospective rasterstereographic pilot study. Eur. Spine J..

[39] Ohlendorf D, Mickel C, Filmann N, Wanke EM, Groneberg DA (2016). Standard values of the upper body posture and postural control: A study protocol. J. Occup. Med. Toxicol. (London, England).

[40] Maurer-Grubinger C, Avaniadi I, Adjami F, Christian W, Doerry C, Fay V (2020). Systematic changes of the static upper body posture with a symmetric occlusion condition. BMC Musculoskelet. Disord..

[41] https://www.euro.who.int/en/health-topics/disease-prevention/nutrition/a-healthy-lifestyle/body-mass-index-bmi?source=post_page---------------------------&msclkid=d769a880d03211eca638246b855e3c4a (Acessed 10.05.2022) WHOEBMI-B.

[42] Sheeran L, Hemming R, van Deursen R, Sparkes V (2018). Can different seating aids influence a sitting posture in healthy individuals and does gender matter?. Cogent Eng..

[43] Kuo YL, Tully EA, Galea MP (2009). Video analysis of sagittal spinal posture in healthy young and older adults. J. Manip. Physiol. Ther..

[44] Kuo YL, Tully EA, Galea MP (2010). Kinematics of sagittal spine and lower limb movement in healthy older adults during sit-to-stand from two seat heights. Spine.

[45] Betsch M, Michalik R, Graber M, Wild M, Krauspe R, Zilkens C (2019). Influence of leg length inequalities on pelvis and spine in patients with total hip arthroplasty. PLoS ONE.

[46] Ackermann H (1983). Sind, “x±2s”-Bereiche nützliche diagnostische Hilfsmittel?. Med Welt.

[47] Ackermann H (1985). Mehrdimensionale Nicht-Parametrische Normbereiche–Methodologische und medizinische Aspekte.

[48] Lüpsen, H. Varianzanalysen—Prüfen der Voraussetzungen und nichtparametrische Methoden sowie praktische Anwendungen mit R und SPSS. https://www.uni-koeln de/~luepsen/statistik/texte/nonpar-anova pdf (Access: 24082022). (2019).

[49] Statista. Bevölkerung in Deutschland nach Häufigkeit des Sporttreibens in der Freizeit von 2017 bis 2021. https://de.statista.com/statistik/daten/studie/171911/umfrage/haeufigkeit-sport-treiben-in-der-freizeit/ (Access: 03.01.2022).

[50] Padulo J, Ardigo LP (2014). Formetric 4D rasterstereography. Biomed. Res. Int..

[51] Mensink GBM, Schienkiewitz A, Haftenberger M, Lampert T, Ziese T, Scheidt-Nave C (2013). Übergewicht und Adipositas in Deutschland. Bundesgesundheitsblatt Gesundheitsforschung Gesundheitsschutz.

[52] https://www.destatis.de/DE/Themen/Gesellschaft-Umwelt/Gesundheit/Gesundheitszustand-Relevantes-Verhalten/Tabellen/koerpermasse-maenner.html (25.03.2020).

[53] https://de.statista.com/statistik/daten/studie/256578/umfrage/bevoelkerungsanteile-in-deutschland-nach-koerpermassen-bmi-und-altersgruppen/ (25.03.2020).

[54] Celan D, Palfy M, Bracun D, Turk Z, Mozina J, Komadina R (2012). Measurement of spinal sagittal curvatures using the laser triangulation method. Coll. Antropol..

[55] Singh DK, Bailey M, Lee R (2010). Biplanar measurement of thoracolumbar curvature in older adults using an electromagnetic tracking device. Arch. Phys. Med. Rehabil..

[56] Zappalá M, Lightbourne S, Heneghan NR (2021). The relationship between thoracic kyphosis and age, and normative values across age groups: A systematic review of healthy adults. J. Orthop. Surg. Res..

[57] Hirschfeld HP, Kinsella R, Duque G (2017). Osteosarcopenia: Where bone, muscle, and fat collide. Osteoporos. Int. J. Establ. Result Coop. Between Eur. Found. Osteoporos. Natl. Osteoporos. Found. USA.

[58] Hüther-Becker, A. editor. Das neue Denkmodell in der Physiotherapie Band 2: Bewegungsentwicklung und Bewegungskontrolle 1ed. Stuttgart: Thieme Georg Verlag (2005).

[59] Laube W, von Heymann W (2012). Das sensomotorische system und die Auswirkungen der physiologie des Alterungsprozesses. Man. Med..

[60] Aagaard P, Suetta C, Caserotti P, Magnusson SP, Kjær M (2010). Role of the nervous system in sarcopenia and muscle atrophy with aging: Strength training as a countermeasure. Scand. J. Med. Sci. Sports.

[61] Chiba R, Takakusaki K, Ota J, Yozu A, Haga N (2016). Human upright posture control models based on multisensory inputs; In fast and slow dynamics. Neurosci. Res..

[62] Kado DM (2009). The rehabilitation of hyperkyphotic posture in the elderly. Eur. J. Phys. Rehabil. Med..

[63] Roussouly P, Gollogly S, Berthonnaud E, Dimnet J (2005). Classification of the normal variation in the sagittal alignment of the human lumbar spine and pelvis in the standing position. Spine.

[64] Katzman WB, Wanek L, Shepherd JA, Sellmeyer DE (2010). Age-related hyperkyphosis: Its causes, consequences, and management. J. Orthop. Sports Phys. Ther..

[65] Ku PX, Abu Osman NA, Yusof A, Wan Abas WAB (2012). Biomechanical evaluation of the relationship between postural control and body mass index. J. Biomech..

[66] Onyemaechi NO, Anyanwu GE, Obikili EN, Onwuasoigwe O, Nwankwo OE (2016). Impact of overweight and obesity on the musculoskeletal system using lumbosacral angles. Patient Prefer. Adherence.

[67] Gong H, Sun L, Yang R, Pang J, Chen B, Qi R (2019). Changes of upright body posture in the sagittal plane of men and women occurring with aging—A cross sectional study. BMC Geriatr..

[68] Decker S, Müller C, Omar M, Krettek C, Schwab F, Trobisch P (2015). Sagittal balance of the spine–clinical importance and radiographic assessment. Zeitschrift fur Orthopadie und Unfallchirurgie.

[69] Porto AB, Okazaki VHA (2018). Thoracic kyphosis and lumbar lordosis assessment by radiography and photogrammetry: A review of normative values and reliability. J. Manip. Physiol. Ther..

[70] Yukawa Y, Kato F, Suda K, Yamagata M, Ueta T, Yoshida M (2018). Normative data for parameters of sagittal spinal alignment in healthy subjects: An analysis of gender specific differences and changes with aging in 626 asymptomatic individuals. Eur. Spine J. Off. Publ. Eur. Spine Soc. Eur. Spinal Deform. Soc. Eur. Sect. Cerv. Spine Res. Soc..

[71] Conder R, Zamani R, Akrami M (2019). The biomechanics of pregnancy: A systematic review. J. Funct. Morphol. Kinesiol..

